# The Effectiveness of Rehabilitation Interventions on the Employment and Functioning of People with Intellectual Disabilities: A Systematic Review

**DOI:** 10.1007/s10926-019-09837-2

**Published:** 2019-05-16

**Authors:** Nina Nevala, Irmeli Pehkonen, Antti Teittinen, Hannu T. Vesala, Pia Pörtfors, Heidi Anttila

**Affiliations:** 1 Finnish Institute of Occupational Health, PO Box 40, 00032 Työterveyslaitos, Finland; 2 The Finnish Association On Intellectual and Developmental Disabilities, Viljatie 4 A, 007004 Helsinki, Finland; 3 National Institute for Health and Welfare, PO Box 30, 00271 Helsinki, Finland

**Keywords:** Employment, Intellectual Disability, Developmental Disability, Learning Disability, Rehabilitation, Systematic review, Barriers, Facilitators

## Abstract

*Purpose* This systematic review analyzed the effectiveness of rehabilitation interventions on the employment and functioning of people with intellectual disabilities (ID), as well as barriers and facilitators of employment. *Methods* This was a systematic review of quantitative, qualitative, and mixed methods studies. The outcomes were employment, transition to the open labor market and functioning. The review included qualitative studies of employment barriers and facilitators. The population comprised people with ID aged 16–68 years. Peer-reviewed articles published in English between January 1990 and February 2019 were obtained from the databases Cinahl, the Cochrane Library, Embase, Eric, Medic, Medline, OTseeker, Pedro, PsycInfo, PubMed, Socindex, and the Web of Science. We also searched Google Scholar and Base. The modified selection instrument (PIOS: participants, intervention, outcome, and study design) used in the selection of the articles depended on the selection criteria. *Results* Ten quantitative (one randomized controlled, one concurrently controlled, and eight cohort studies), six qualitative studies, one multimethod study, and 21 case studies met the inclusion criteria. The quantitative studies showed that secondary education increases employment among people with ID when it includes work experience and personal support services. Supported employment also increased employment in the open labor market, which sheltered work did not. The barriers to employment were the use of sheltered work, discrimination in vocational experience, the use of class teaching, and deficient work experience while still at school. The facilitators of employment were one’s own activity, the support of one’s family, job coaching, a well-designed work environment, appreciation of one’s work, support form one’s employer and work organization, knowledge and experience of employment during secondary education, and for entrepreneurs, the use of a support person. *Conclusions* The employment of people with ID can be improved through secondary education including proper teaching methods and personal support services, the use of supported work, workplace accommodations and support from one’s family and employer. These results can be utilized in the development of rehabilitation, education, and the employment of people with ID, to allow them the opportunity to work in the open labor market and participate in society.

**Electronic supplementary material:**

The online version of this article (10.1007/s10926-019-09837-2) contains supplementary material, which is available to authorized users.

## Background

Employment is one of the primary goals of people with intellectual disabilities (ID) [1, 2]. Employment can lead to positive psychosocial and economic benefits for people with ID, including a sense of purpose, opportunities for new friendships [1, 2], health [3] and better quality of life [4, 5].

People with ID seldom work in the open labor market and the proportion of people with ID in employment varies between countries. For example, in Finland, it is 3% of working-age people with ID [6], in England 5–11% [7–10], and in the USA, 10% [11].

The prevalence of ID is about 1% of the population, but it differs between countries [12]. In Finland, it is 1% [13, 14], as in most European countries [15]. In Finland, 0.8% of working-aged people have ID, which means 25,000 people [6].

Rehabilitation is a goal-oriented process, which here aims to enable people with ID to reach an optimum mental, physical and social functional level, thereby providing them with the tools required to change their lives [16]. People with ID should receive services that support their functioning, self-determination and participation [2, 17]. In the last years, instead of using a system-centered approach, services for people with ID and other disabilities have begun to use a person-centered approach, tailoring services around the individual rather than enforcing a one-size-fits-all principle [2, 18, 19]. According to Austin and Lee [20], job-related services (job search assistance, job placement, job readiness training, on-the-job support services) significantly predicted the employment outcomes of people with ID. However, they found no significant relationship between personal services (diagnosis and treatment, counseling and guidance, transportation, maintenance, miscellaneous training) and employment outcomes.

Sheltered work or workshops are one form of rehabilitation for people with ID. One of the main tenets often cited by supporters of sheltered workshops is that center-based programs provide employment opportunities for people with ID in a segregated environment by building up their vocational and social skills [21, 22]. However, young adults with mild to moderate ID also have difficulties transitioning from sheltered workshops to the open labor market. Moreover, parents’ overprotection, important friendships, and the attitudes of employers and co-workers can be barriers during this transition process [22].

In this review, the term intellectual disability (ID) refers to people who have a significantly reduced ability to understand new or complex information and to learn new skills (impaired intelligence), and who have reduced ability to cope independently (restricted social functioning). In the United Kingdom (UK) the term ‘people with learning disabilities’ (LD) is used to describe those referred to elsewhere as people with ‘intellectual disabilities’ or ‘developmental disabilities’ [23].

No reviews were found on the effects of rehabilitation on employment among people with ID. Some reviews, however, evaluated the effectiveness of rehabilitation on the functioning (activities of daily living, self-care skills, communication skills, and cognitive achievements) of people with ID [16], the effects of the person-centered planning of services for people with ID on different outcomes (i.e., employment) [17], and effects of technology use on employment [24]. The research questions were: 1) How effective are rehabilitation interventions for the employment of people with ID, 2) what are the barriers to and facilitators of employment of people with ID, and 3) what kind of individual support measures have been used to increase the work ability and functioning of people with ID?

**Fig. 1:**
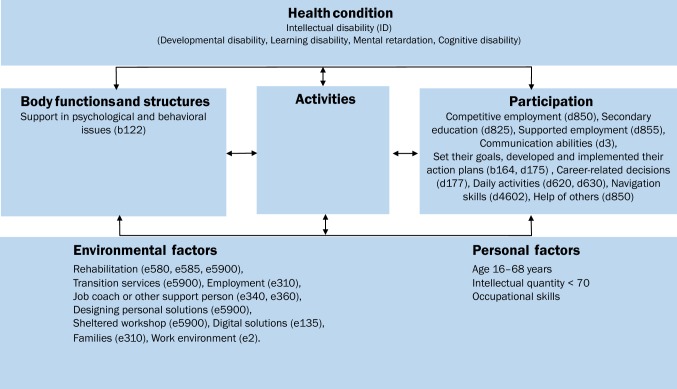
The theoretical orientation that guided this study was the ICF (International Classification of Functioning, Disability and Health) framework [25]

The theoretical orientation guiding this study was the International Classification of Functioning, Disability and Health (ICF) framework [25] (Fig. 1). The ICF identifies and classifies the domain of environmental factors, including rehabilitation, as one of its health-related domains [26–28]. ICF provides a detailed framework for describing disease experiences as a dynamic interaction between its components, and ‘core sets’, comprising lists of ICF categories [28]. The ICF identifies and classifies the component of environmental factors, including rehabilitation, as one of its health-related components [26, 27]. According to the ICF, these environmental factors can also be either barriers to or facilitators of employment for these individual [27].

## Methods

This systematic review analyzed the effectiveness of rehabilitation interventions on the employment and functioning of people with ID, as well as barriers and facilitators of employment. We included quantitative, qualitative, and multimethod studies, because they provided different knowledge regarding this phenomenon [29, 30]. Quantitative studies showed the effectiveness of rehabilitation; qualitative studies showed the process, facilitators and barriers; and multimethod studies combined these.

We used the modified selection instrument (PIOS: participants, intervention, outcome, and study design) [29, 31] to select the titles, abstracts, and full papers according to the selection criteria. The inclusion criteria for studies were randomized controlled trials (RCTs) and non-randomized studies such as concurrently controlled trials (CCTs), case–control studies, cohort studies, follow-up studies, and case studies that investigated the effectiveness of rehabilitation among people with ID. We expected the outcomes of the studies to be employment (supported or non-supported employment, transition from school to work, transition from sheltered workshop to employment), or functioning (job performance), and the population to comprise people with ID (developmental disabilities, learning disabilities, cognitive disabilities, mental retardation, mental handicaps), in the age group of 16–68 years.

We searched for articles published in English from January 1990 to February 2019. The databases we searched in February and October in 2016 and in February in 2019 included: Cinahl, the Cochrane Library, Embase, Eric, Medic, Medline, OTseeker, Pedro, PsycInfo, PubMed, Socindex and the Web of Science. In addition, we searched Google Scholar and Base. Our main search terms were: (1) intellectual disability, mental disability, mental retardation, mentally disabled persons; (2) employment, employability, sheltered work, work capacity, work ability, vocational status, career; (3) rehabilitation, treatment, therapy, training, career counseling, social support, education, program, intervention, assistive technology, counseling, mainstreaming; (4) outcomes, outcome assessment, randomized controlled trials, program evaluation, effectiveness, validation studies, evaluation studies, comparative studies, cost–benefit analysis, before-after and follow up. We also manually scanned reference lists of identified reviews for additional relevant articles. Full details of the search strategy are available (Supplementary material).

The review team comprised five researchers, whose areas of expertise were disability, ergonomics, rehabilitation, social sciences, interventions, systematic reviews, and quantitative and qualitative methodology. First, pairs of researchers independently reviewed titles and abstracts and made a unanimous decision. If consensus was not reached, a third researcher was consulted. The full texts of all the eligible articles and those whose eligibility could not be discerned from reading the abstract were obtained.

Two reviewers independently assessed the methodological quality of the included RCT and CCT studies using the checklist by van Tulder et al. [32], which has also been used in other reviews concerning rehabilitation [28, 33, 34] and workplace accommodation [35]. The checklist consisted of 11 criteria. Each item scored two points for ‘Yes’, one point for ‘Don’t know’, and 0 points for ‘No’. The item scores were summed into a single total score (range 0–22). Studies were considered to be of high methodological quality if the score was at least 11 out of 22, and of low methodological quality if they achieved fewer than 11 points [32].

The cohort studies and the mixed method study were assessed using the Newcastle–Ottawa Scale (NOS) [36]. This assessment scale consists of eight items within the following three categories: selection of study groups (four items), comparability of groups (one item/two questions) and ascertainment of exposure/outcome (three items). The highest value for quality assessment is nine ‘stars’. One star is allocated for each item in the selection and outcome categories and two stars in the comparability category. Study quality was graded as follows: 1–3 stars (low quality), 4–6 stars (intermediate quality), and 7–9 stars (high quality) [36].

Three pairs of reviewers independently assessed the methodological quality of the included qualitative studies using a modified version of the CASP method [37]. Any difference in the item scoring was resolved through discussion with the third author until consensus was achieved. This assessment tool consisted of evaluation criteria that are commonly acknowledged as crucial in qualitative research in the social sciences. The assessment tool involved 10 questions based on the following four main principles: The research should (1) provide a defensible research strategy that can answer the questions posed, (2) demonstrate rigor through systematic and transparent data collection, analysis and interpretation, (3) contribute to advancing wider knowledge and understanding, and (4) demonstrate credibility with plausible arguments about the significance of the findings. Each item scored ‘Yes’ or ‘No, depending on whether the topic was described sufficiently. An additional score of ‘Partially’ was added, as the original checklist was not able to adequately differentiate between the quality of the studies [38]. This addition resulted in three options: ‘Yes’ (2 points), ‘Partially’ (1 point), and ‘No’ (0 points). The higher the total score, the better the methodological quality, with a maximum score of 20. The studies were rated as being of high methodological quality if they achieved more than 10 points.

The case studies were assessed using eight questions from the Joanna Briggs Institute (JBI) method [39]: (1) Were the demographic characteristics of the person attending clearly described? (2) Was the history of the person attending clearly described and presented in the timeline? (3) Was the current clinical condition of the person clearly described, (4) Were diagnostic tests or assessment methods and the results clearly described? (5) Was the intervention(s) or treatment procedure(s) clearly presented? (6) Was the post-intervention clinical condition clearly described? (7) Were adverse events (harms) or unanticipated events clearly described? (8) Does the case report provide takeaway lessons? Each question scored one point for ‘Yes’, two points for ‘No’, three points for ‘Unclear’, and four points for ‘Not applicable’.

All the reviewers participated in the data extraction, which was carried out separately to the quantitative, qualitative, and multimethod studies, and included details on the participants and descriptions of the rehabilitation and the outcomes. The meaningful concepts of each outcome were linked to the ICF categories [40, 41].

We descriptively synthesized the data using tables to describe the characteristics, results and quality of the included studies. Different tables were used to describe the quantitative, qualitative and case studies. We categorized the overall quality of the quantitative studies and their outcomes using the principles of GRADE (Grading of Recommendations, Assessment, Development, and Evaluation) [42]. The GRADE approach classifies the quality of evidence as high, moderate, low or very low. Randomized controlled studies’ evidence begins with strong and cohort studies with scant evidence on the basis of the assumption that randomization is the best method for controlling unknown factors. Five factors can weaken evidence: (1) the risk of bias, which results from the weakness of the research method and its implementation, (2) differences and inconsistencies between different studies, (3) indirectness of the results in comparison to the PICO criteria, (4) the inaccuracy of the results (the number of events and participants and their effect on confidence intervals) and (5) publication bias (Chapter 12, Cochrane Handbook). Thus, strong research evidence required at least two-high quality studies, the results of which were parallel. Moderate research results required one or several high-quality studies, the results of which were only slightly contradictory, or several adequate studies, the results of which were parallel. Scant research evidence meant that the research results had significant contradictions. No research evidence meant that no studies existed or that they were methodically weak.

## Results

**Fig. 2:**
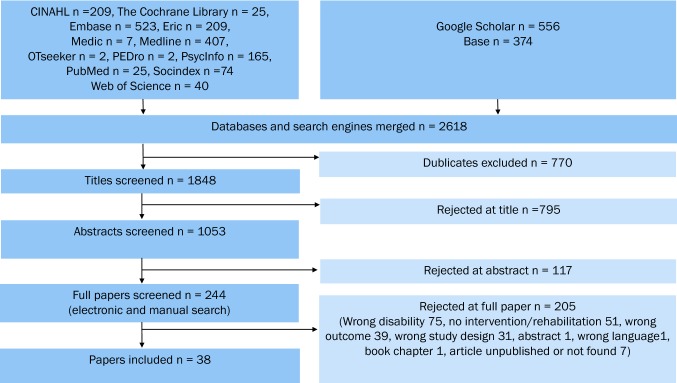
Flow chart of review identification and selection process

The search strategy identified 2618 references (Fig. 2). After removing duplicates, 1848 references remained. Two of the reviewer (altogether five) authors scrutinized all the titles and abstracts according to the inclusion criteria, and when information necessary for inclusion was lacking, they read the articles’ full texts. We obtained the full texts of 244 articles, and 38 studies met our inclusion criteria.

### Characteristics of the included studies

#### Design and methods

Ten of the 38 included studies were quantitative (Table 1), six studies were qualitative (Table 2), one was a mixed methods study (Table 2), and 21 were case studies (Table 3, 4). The quantitative studies included one RCT [43], one CCT [44] and eight cohort studies [45–52]. The qualitative studies included three interview studies [53–55], one interview and document study [56] and two ethnographic studies [57, 58]. The mixed method study [59] used structured interviews, focus group interviews and register analysis. The 21 case studies included five qualitative case studies [60–64] and 16 quantitative experiments [65–80].

**Table 1:** Characteristics of included quantitative studies

Authors, year, country	Study design, methods	Population, definitions/terms	Intervention/rehabilitation/personally tailored/length of intervention/personal or environmental measures	Outcome measures	Findings
Arvidsson et al. [50], Sweden	A cohort study. Regression analysis of register data from Halmstad University Register on Pupils with Intellectual Disability (based on complete national survey of students who graduated from Upper Secondary School for Pupils With Intellectual Disability in Sweden) merged with data from two national registers, the Integrated Database for Labor Market Research (LISA; which includes all people in Sweden over the age of 15) and the LSS register (which includes all those who receive services under the Act Concerning Support and Service for Persons with Certain Functional Impairments)	N = 2745 (71% M/29% F) gainfully employed pupils with intellectual disability (22.4% of 12,269 students who graduated from Upper Secondary School for Pupils with Intellectual Disability between 2001 and 2011)	Upper Secondary School for Pupils with Intellectual Disability programs: national program, specially designed program, vocational training, training activities, or graduated with incomplete grades	Gainful employment i.e. work full- or part-time and have either unsubsidized or subsidized employment (they all had a job during 2011 that generated a salary reported to the Swedish tax agency and they neither had daily activities according to LSS nor were registered as students. The variable gainful employment is dichotomous and states whether a person was gainfully employed or not in 2011, according to the LISA register	339 people (72% M/28% F) were gainfully employed without any type of subsidies. 195 (57.5%) had attended a Upper Secondary School for Pupils With Intellectual Disability programs, 90 (26.5%) attended a specially designed program, 46 (13.6%) graduated from USSID with incomplete grades, and 8 people (2.4%) attended an individual program/vocational training Of the 96 women, 70.8% worked in the private sector and 24% in the public sector. The most frequent occupation (36.5%) was “personal care and related work.” The 243 men worked primarily in the private sector (93.4%), with the largest proportion (18.9%) in professions described as “other service work” (e.g. janitors, garbage collectors, newspaper and package deliverers) Probability of being gainfully employed: OR: 3.84 national program, OR: 2.87 specially designed program, and OR: 0.31 vocational training program, compared to those who graduated with incomplete grades. Men (OR: 4.06) were more likely to be employed than the women (OR: 3.51)
Beyer and Kaehne [46], UK	A cohort study. Carried out in six local authority areas in England, Scotland and Wales. A total of 14 special schools took part in this study. Logistic regression	N = 87 (61%M/39%F), Dg: LD, additional diagnoses/problems: Autistic spectrum disorder (9%), Asperger’s syndrome (1%), emotional or behavioral difficulties (5%) IQ: nr Age:17.8 years Occupation: nr	Work awareness training (e.g. watching videos showing work, talking about presenting one’s self at work, health and safety instruction), vocational qualifications courses, work experience (In special schools provided within the school e.g. assisting in class for younger children, helping the janitor, working in the kitchen and in external transition organizations primarily gained in the community with or without a job coach), vocational profiling, practical project placement (e.g. café, sandwich-making, and furniture-making enterprises)	Full- or part-time employment six month post graduation	Employment 18 (21%) of the young people were employed (56% from mainstream schools, 33% from colleges and 11% from special schools). 2 variables were significantly related to employment. Persons that had work experience (provided by external transition support organizations) were 1.01 times more likely to be employed, and those that had work awareness training (provided by the schools/colleges) were also 1.01 times more likely to be employed. These 2 variables explained 19.2% of the variance in predicting employment post graduation
Bouck and Chamberlain [51], USA	Secondary data analysis of the National Longitudinal Transition Study-2 (NLTS2). The data collection occurred from 2000 to 2009. This study included postschool data, including postschool services received and postschool outcomes. Survey via telephone, mailed survey. Frequency counts, F tests, cross-tabulations, logistic regression analyses	N = 121,335 Age: 20.2 years (17–25). Gender: nr. Dg: mild ID. IQ: nr. Ethnicity: White 66.7%, African American 23.6%, Hispanic 8.8%, Asian/Pacific Islander 0.1%	Postschool services: postsecondary education institution accommodations and services, job training services, and life skills services	Paid employment, full-time employment	Significantly more (p < .0.1) individuals who did not receive job training services were employed full-time (62.7%) compared with those who received job training services (16.5%). No significant differences were between those who did (56.2%) or did not (65.3%) receive job training services with regard to paid employment
Cimera [47], USA	Cohort study, two cohorts, matched pairs 2002–2006. Data comes from the Rehabilitation Services Administration’s 911-database. Two groups of supported employees were compared	N = 9808. Sheltered workshops cohort: N = 4904 (41.7% F/58.3% M). Age: 38.93 years. Not in sheltered workshops cohort: N = 4904 (41.7% F/58.3% M). Age: 31.56 years	Sheltered workshops	The rate of competitive employment	Employment There was no difference in the rates of employment for these two groups. 59.6% of sheltered workshop supported employees were competitively employed compared to 60.4% for individuals who did not participate in shetered workshops prior to entering supported employment. This difference was not statistically significant
Cimera et al. [49], USA	Cohort study. Matched pairs 2006–2009. Two groups were compared	N = 15,040 (7520 in both groups) (54.6–56.7% M/43.3–45.4% F). Age (average): 20.28–20.33 years. Dg: Significant ID. Multiple disability 6.7–13.6%. IQ: nr. Ethnicity: White 46.8–50.6%, African American 48.5–52.3%, Asian 0.3–0.75, Native American 0.4–0.5%, Pacific Islanders < 0.1%, Hispanic 2.4–3.7%. Occupation: nr	Transition services. Early transition group: transition services were addressed in individualized educational programs by age 14. Later transition group: transition services were addressed by age 16	Successful employment (e.g. employment in the community paying at least minimum wage)	Employment Individuals from the early transition states were significantly more likely to become employed than their matched peers from the later transition states. For example, in 2006, 74.3% of young adults from the early transition states were employed compared with 57.8% for the later transition states
Goldberg et al. [43], USA	A randomized controlled trial (RCT). A Functional Assessment Scale. Chi square. Spearman correlation coefficient	N = 49 (M 24/F 25), experimental group N = 24, control group N = 25 Age: mean 31.2 years (experimental group), 30 years (control group) Dg: DD since birth	Experimental group (supported employment program): placement agents who made contacts with employers in the community, clients were immediately prepared for competitive employment with a variety of supportive services (i.e., job monitoring, social work with families). Control group (conventional workshop services): placement agents who made contacts with employers in the community, staff waited until clients were considered ready before placing them outside the workshop	Employment in the competitive labor market (employed for 60 days) and in three categories: no work, some work (work at least 2 weeks), and currently working. Performance (Functional Assessment Scale)	Employment Persons in the experimental group (supported work program) had a better change of obtaining employment than the control group X2 (2, N = 49) = 8.5, p < 0.02. of 24 persons in the experimental group, 20.8% were currently working, and 8.3% worked at some time. None of the control group had worked in competitive employment Performance There were no significant differences in performance between the groups. Pretest/posttest results were in the experimental group 54.2 (6.4)/51.4 (8.0), and in the control group 53.3 (7.6)/53.6 (7.4)
Gray et al. [45], USA	Cohort study. The data were provided by a State agency (South Carolina Department of Disabilities and Special Needs) and by the local boards. Registers, written survey. Logistic regression	Employed in 1997: N = 431, 59.6%M/40.4%F, Age: 19–30 years 44.3%, 31–50 years 49.0%, 51–64 years 6.7%. Ethnicity: White 42.5%, nonwhite 57.5% IQ: 20–29 3.7%, 30–39 15.1%, 40–49 20.2%, 50–59 24.8%, 60–69 31.6%, 70–74 4.6% Unemployed in 1997: N = 6659, 48.7% M/45.3%F. Age: 19–30 years 35.1%, 31–50 years 51.3%, 51–64 years 13.7% Ethnicity: White 51.9%, nonwhite 48.1% IQ: 20–29 16.5%, 30–39 24.9%, 40–49 14.3%, 50–59 17.8%, 60–69 22.2%, 70–74 4.3%	Number of job coaches	Employment (earning at least $50 per week in a competitive job or enclave for at least six months in 1997)	Employment The number of coaches varied from 0.00 to 2.48 per 100 individuals. Most individuals (82.6%) were served between 0.5 to 1.5 job coaches per individuals. Significant, positive effects of job coaches on employment were found as follows: The risk of employment as a function of coaches was approximately two times greater for individuals with low than for high IQ, approximately three times greater for individuals in counties with low or intermediate employment, and approximately ten times greater for individuals located in highly urban counties
Joshi et al. [48], USA	Cohort study. A study utilized data collected in the National Longitudinal Transition Study-2. Telephone interview, mail survey. Descriptive analysis, frequency counts, multiple regression and logistic regression analysis	N = 62,513 (61.7%M, 38.3%F). Age: Average age for students in school was 18 years, average age for students out of school was 20.1 years. Dg: Mild ID. Ethnicity: Caucasian 70.2%, African American 24%, Hispanic 5.2%	Students received special education services between 13 and 16 years of age. Employment-related transition activities identified in NLTS2 data were vocational assessment, career counseling, prevocational education, career technical education or vocational education, prevocational or job readiness training, instruction looking for jobs, job shadowing, job coach, special job skills training, placement support, internship or apprenticeship programs, tech prep programs, work experiences in school, and other paid work experiences	Postschool employment (e.g. whether they ever engaged in employment, their current employment status, and whether they worked part- or full-time)	75.9% of the students reported they engaged in employment sometime after leaving school. 62.1% were currently employed and 42.6% were engaged in full-time employment. Participation in one additional transition activity represented within the employment activities summation variable resulted in students being 1.2 times likely to be currently employed post school. Students were 5.704 times as likely to ever engage in employment after school if they engaged in paid-employment experiences while in school, and 3.489 times as likely to ever engage in employment after school if they experienced a school-sponsored job. However, vocational education was not found to be significantly related to postschool employment
Kilsby and Beyer [44], UK	Intervention study. An independent groups design (a baseline and two intervention phases, three months each). Direct observations, questionnaire, taster sessions, job review forms. A one-way ANOVA	N = 35. Three groups matched. Baseline: N = 12 (M 58%/F 42%). Age: 32 years (mean). Dg: 58% ID borderline, 33% mild, 8% moderate Intervention 1: N = 12 (M 50%/F 50%) Age: 26 (mean) Dg: 22% borderline, 45% mild, 33% moderate Intervention 2: N = 11 (M 50%/F 50%) Age: 32 (mean) Dg: 54% borderline, 38% mild, 6% moderate	A job taster program (a two phase self-determination package) for adults with LD entering the job market for the first time. Job tasters were short, unpaid, time-limited work experiences which took place in the workplace in order to allow people to sample a variety of different jobs and work cultures. In each group, each participant was required to attend 3 job tasters for 6 sessions. Baseline: No specific instructions were provided to agency staff about how to conduct the job tasters. Intervention 1: Introduction to self-determination and systematic job taster reviews. A one-day package was implemented to introduce job coaches to the concepts of self-determination using Wehmeyer’s definition as a conceptual base; and job reviews were introduced. Job reviews were short meetings where job seekers were encouraged to evaluate the job taster sessions and overall program. Intervention 2: Introduction of pictorial job review profile. A second one-day package was implemented: job coaches were encouraged to support the job seekers to complete the job review forms for themselves. In order to help those who could not read, a pictorial questionnaire replaced the written version	The rate of overall job coach assistance (instructions and questions). An average rate of assistance per minute was calculated by dividing the frequency of job coach assistance by the duration in minutes	Performance Intervention 1 led to a reduction in rates of job coach assistance for both sessions and reviews. The rate of assistance fell significantly between Baseline (0.83 per min) and Intervention 1 (0.51 per min). The introduction of intervention 2 led to a further drop in rates of assistance during the reviews. The reviews in intervention 1 and 2 were generally accurate, and job seekers were consistent with their statements. This indicate that the job seekers had an awareness of their surroundings and a realistic grasp of their personal limitations and abilities
Sannicandro et al. [52], USA	A secondary data analysis of the RSA-911 datasets from 2008 through 2013. A quasi-experimental design Multilevel modeling, propensity score matching techniques. Multilevel logistic regressions and multilevel linear regressions, depending on whether the outcome was binary or continuous	N = 11,280. Experimental group: N = 5640, (M 46.9%/F 53.1%). Age: 20.9 years. Dg: ID. Ethnicity: White 57.6%, Black 31.1%, American Indian 1.5%, Asian 2%, Native Hawiian or Pacific Islander 0.5%, Hispanic 7.3%. Control group: N = 5640, (M 57.2%/F 42.8%). Age: 20.4 years. Dg: ID. Ethnicity: White 52.6%, Black 36%, American Indian 1.3%, Asian 1.4%, Native Hawiian or Pacific Islander 0.4%, Hispanic 8.3%	Postsecondary education was comprised of positive response to the following variables from the RSA-911 dataset (1) postsecondary education with no degree, (2) associate degree or vocational/technical certificate, (3) bachelor’s degree or, (3) master’s degree or higher	Employed at closure (yes/no)	Individuals who participated in postsecondary education were more likely to be employed (OR: 2.00, 95% CI:1.82–2.20) than their counterparts who had not participated in postsecondary education

*N* number, *M* males, *F* females, *Dg* diagnosis, *IQ* Intellectual Quantity, *nr* not reported, *ID* Intellectual Disability, *IDD* Intellectual and Developmental Disability, *DD* Developmental Disability, *LD* Learning Disability, *MR* Mental Retardation, *SE* Supported Employment

**Table 2:** Characteristics of included qualitative studies and a multimethod study

Authors, year, country	Methods, study design	Population, definitions, terms	Intervention	Findings, barriers and facilitators
Alborno and Gaad [56], Dubai	Observation, semi-structured interviews, document analysis. Data triangulation	N = 27 (100% M) Age: 19–37 years Dg: ID and physical disabilities (nine with Down syndrome) IQ: nr. Average mental age 4–15 years. Most had attended rehabilitation centers where they had learned some basic life skills	Employment either in administrative jobs in the head office or as gardeners in the nursery. Functional and vocational training programs, productivity monitoring, recreational and sports activities, support in psychological and behavioral issues by documenting the development of the behavioral, psychological, and professional skills of the employees with disabilities, and regular provision of individualized and group sessions for the employees to address any behavioral or psychological issues, and transportation services in dedicated buses supplied for the group	All were successfully and fully integrated into their jobs: data entry, public relations, photography, and office mail distribution. Among their peers and the company’s management, they were recognized as loyal, efficient, and reliable staff The employer recognized that certain barriers between their employees with disabilities and the work environment had to be overcome The employees felt they were valued and respected, which resulted in higher self-esteem and self-confidence. They were also trusted through an open-door policy with management which allowed them to voice their opinions and needs. High job satisfaction *Facilitators* Recruitment with no formal testing of abilities Training: efficient, staged training programs Workplace accommodations (shading the area from the sun, shorter working hours in summer, fully paid two-month summer holiday, special chairs, modified equipment, absence allowed for rehabilitation/treatment, first aid training workshops, transportation services with company busses) Management, supervision, and performance monitoring (support of staff and management, behavioral tally sheets, positive behaviors such as greeting peers, cooperation, following orders, and speech) as well as negative behaviors (such as rude words, violence, disobedience, or stubbornness) were recorded, which enabled early detection of problems and effective problem-solving involving all stakeholders Incentives such as adequate remuneration, professional development, and recreational and social programs resulted in empowerment and positive attitudes, creating a friendly work environment Professional progress: opportunities for developing new skills
Christensen and Richardson [55], USA	Semi-structured interviews. Inductive content analysis approach	N = 10 (M = 6) Age: 25–51 years Dg: ID IQ: nr	Project SEARCH is a licensed transition-to-work model, which features total workplace immersion, and facilitates a seamless combination of classroom instruction, career exploration, and on-the-job training and support. The goal was the transition of individuals out of sheltered workshops to competitive employment	Eight persons completed the course and five of them were employed. The program emphasized learning about other job opportunities in the community (e.g., touring local businesses). Individuals with significant disabilities can imagine themselves working in the community when they are exposed to a variety of career opportunities and can directly see and under-stand what the job entails The cohort model of Project SEARCH allowed ten individuals to exit the workshop and transition into a community setting with familiar peers. This alleviated some of the initial fears of losing friends as a result of leaving the workshop. During the course the participants became more independent and lived in the community, and their views about the meanings of employment changed. Participants demonstrated an increased awareness of employment as a means to take greater responsibility as a productive member of their house-hold and the community. Competitive employment represented the means to earn wages in order to pay their own bills and not have to rely on Medicaid benefits The sample size is small and localized. While the results of this study provide some level of insight regarding the experiences of individuals with IDD who are transitioning from a sheltered workshop into competitive, integrated employment, the views expressed by program participants are limited to their own unique experiences
Devlieger and Trach [57], USA	Ethnographic study. Extensive life history interviews with focal people, parents, and agency or school personnel	N = 6 (M = 4) Age: 18–24 years Dg: Mild mental retardation IQ: nr	Intervention not described, instead the impact of different actors (school, agency, parents, other people) on the transition process and employment was analyzed	The involvement of parents and focal people was disproportionate to that of school and agency personnel. School and agency efforts most often resulted in sheltered employment, while personal or parent mediation resulted more often in self-employment and continuing education outcome
Donelly et al. [58], Australia	Ethnographic study. Interviews of participants and their social networks. On average, five people in each network were interviewed. Interviewees included parents, other family members, members of various support circles, support workers, friends and allies. Participant observations were conducted of the three participants’ work or work preparation environments. The fourth participant provided information about her work in interviews	N = 4 (M = 1) Age: 21–48 years Dg: Intellectual disability IQ: nr	No intervention was described	The meaning of work to participants. A range of meanings including experience of meaningful occupation, the development and recognition of skills, experiences such as travelling to and from work, relationships with co-workers, financial remuneration and access to opportunities that extended beyond the workplace The meaning of work to families included skill development, time use (participant’s days filled with meaningful activity), the choices (or interests) of participants as indicators of potential employment opportunities; quality of performance was linked to level of interest; interests and skills were not always valued or recognized. Vocational preparations that failed: formal service providers fitting people into existing programs that did not meet their needs. Classroom-based vocational preparation vs. actual work experiences (failing in classroom may prevent work experience opportunities); Effective vocational preparation: helped the person get a job based on personal interests, skills and choices and provided on-the-job training and support *Barriers to employment* Inadequate identification of support needs Lack of responsiveness to individual needs Emphasis on fitting people into existing support models Loss of vocational skills or failure to develop vocational skills in sheltered employment Difficulty moving from sheltered to competitive employment Classroom-based vocational preparation Use of classroom-based learning experiences and written examinations as pre-requisites for more meaningful, effective employment opportunities Failure of employment agencies to find opportunities for work experience and on-the-job training
Fashing [54], Austria	Grounded theory, biographical interviews	N = 3 (F) Age: nr Dg: ID Women who have similarly difficult post-school vocational orientation phases despite having been successful in their transition to vocational training or employment on the regular labor market	‘What experiences do women with intellectual disabilities have in their transition from school to vocational training and employment?’ The interviewed women initially went from compulsory school to a vocational preparation measure or directly into a sheltered workshop or occupational therap.	Main category: ‘coming to terms with disability through vocational participation’ was formed on the basis of two key categories: discrimination at school due to learning difficulties and post-school orientation phase as a process of self-determination
Winsor et al. [56], USA	Multimethod study. Structured and in-person interviews, focus groups, register data	N = 1452 Participants in PP countries, N = 390 (2008 N = 160, 2009 N = 230) Comparison groups: Non-participants in PP counties N = 656 (2008 N = 315, 2009 N = 341), no PP county students, N = 406, 2008 N = 212, 2009 N = 194) Sex: nr Age: 21 years IQ: nr High school students who were eligible for developmental disability services and had turned 21 during their year of graduation, and participated the project Dg nr. Division of Developmental Disability eligible students. Informants were staff members, stakeholders (county developmental disability staff, school administrators, teachers, employment vendors, family member, and young adults who had obtained jobs)	The starting point of the ‘Jobs by 21 Partnership Project’ (PP) was, that postgraduate employment outcomes seem to be related to employment experiences prior to graduation. The aim of the project was to provide high school students with ID and their families with information about and experiences of employment and the adult service system prior to their graduation, thus making the transition from school to adult life more seamless. The project was implemented in 11 counties (9 in 2008 and 11 in 2009) by local Developmental Disability offices. The local offices gathered different stakeholders to collaborate: Division of Vocational Rehabilitation, the Work Source Center, adult employment vendors, local community colleges, and local businesses Partnership project (PP) goals: 1) a post-school outcome plan for students, 2) expansion and improvement of collaboration in country between Division of Developmental Disability Counties and schools to enable students to make use of available support, 3) establishment of a statewide partnership, 4) to make use of job training and job preparation opportunities, labor market guides, workforce development trends, and post-graduation outcome reports to achieve post-school employment for transition-aged students with developmental disabilities	*Research questions* 1) What was the impact of connecting young adults with employment vendors prior to graduation? 2) How were resources maximized across the school and adult service systems? 3) What strategies were most effective in encouraging collaboration between school and adult service systems? *Results* *Employment outcomes*: PP participants were more likely to be employed following school exit (after three months) and had stronger employment outcomes (mean hours and mean wages) than students who were not participants. In 2008, 45% of participants were employed, compared to 6% of non-participants; in 2009 11% participants were employed, compared to 05% and 5.7% of non-participants *System outcomes:* The availability of project funds encouraged stakeholders from the school and adult service systems to contribute additional dollars and resources in-kind to the project *Collaborative relationships:* The process of leveraging resources helped bring together stakeholders to collectively commit to achieving employment outcomes and problem-solving

*N* number, *M* males, *F* females, *Dg* diagnosis, *IQ* Intellectual Quantity, *nr* not reported, *ID* Intellectual Disability, *IDD* Intellectual and Developmental Disability, *DD* Developmental Disability, *LD* Learning Disability, *MR* Mental Retardation, *SE* Supported Employment

**Table 3:** Characteristics of included quantitative case studies

Authors, year, country	Study design, methods	Population, definitions/terms	Intervention/rehabilitation/personally tailored/length of intervention/personal or environmental measures	Outcome measures	Findings
Allen et al. [76], USA	Quantitative case study. Interrupted time-series design. Observation	N = 3 Ned: M, 18 years, dg: mild MR. Tracey: M, 17 years, dg: moderate MR Emma: F, 16 years, dg: moderate MR	The two interventions (video modeling and audio cuing) were evaluated in an interrupted time series withdrawal design during training and then again in an actual job setting (at the factory and warehouse). During video modeling, the participants watched standard training videos on a laptop computer For the Audio Cuing condition, the participants wore a (Radio Shack TRC-508 s FM) transceiver with microphone and earphones that allowed hands-free operation. During Audio Cuing, the participants were told to ‘Listen to the attendant, who will give you ideas of things you can do to entertain and interact with customers’	Percentage occurrence of multiple skill during 2-min work samples across baseline, video modeling, audio cuing, and 10-min work samples while actually working in the job at 1 and 3 months	Job skills Video modeling was not effective whereas audio cuing produced job performances well above the designated criteria At baseline, Tracey showed a decreasing trend in multiple skills use, Ned showed no evidence of multiple skill use, and Emma showed some evidence of multiple skill use. During video modeling, Tracey showed no evidence of multiple skill use, Ned showed an initial but unsustained increase, and Emma showed a modest increase in multiple skill use. With the introduction of audio cuing, the rates of multiple skills use changed immediately and substantially for Tracey (100%), Emma (90%), and Ned (80%)
Bennet et al. [72], USA	Experimental case study. The design consisted of three conditions including baseline, intervention Covert Audio Coaching (CAC) and follow-up (five consecutive data collection sessions followed by weekly probes. The study was conducted at the participants’ job sites	N = 2 Andy: M, 30 years, dg: ID, IQ: 38 Language summary: speaks in partial sentences in Creole and English Worked as a custodial assistant at a public school Daniel: M, dg: ID, IQ: 55 Language summary: speaks in complete sentences Worked at a food bank	Supported employees received feedback delivered via CAC, which consisted of delivering praise, guidance and correction statements The tasks selected for Andy were washing the windows of automatic sliding glass doors and collecting trash in the schoolyard, and for Daniel stacking bread crates. All the tasks were part of their regular job duties	Work performance (intervention fidelity and impact of CAC on accuracy, durability and fluency): Rate of praise, guidance and corrections delivered to the worker; percentage of task steps completed, pieces collected (in trash collection), rate of correct responses per minute	Work performance Accuracy, durability and fluency: Andy, window washing: baseline < 38%, final sessions 97–100%, slight reduction after intervention. Rate increased by 10.9%. Andy, trash collecting: baseline stable but low, final sessions 66–79%, slight reduction after intervention. Rate increased by 17.4%. Daniel, crate stacking, high but inconsistent, final sessions > 95%, maintained same level after intervention. Rate increased by 78.3% The CAC intervention was effective in increasing the work performance of supported employees. The changes lasted for several weeks after the end of the intervention. The intervention was effective across different participants and work tasks
Carson et al. [71], USA	Case study. Observation	N = 3 Tim: M, 18 years, dg: moderate ID and ADHD Brian: M, 20 years 10 months, dg: moderate ID Hope: F, 20 years 3 months, dg: mild ID	Participants worked using functional skills (e.g., meal preparation) and functional academics (e.g., telling time, using a calculator). The photo activity schedule book (PASB) was used to increase the number of independent task changes in vocational tasks and the rate of vocational task completion. Five color photographs were placed in the album: four showed the individual tasks to be completed and the last depicted the finish location	Number of independent task changes and rate of vocational task completion	Performance The use of the PASB resulted in high levels of independent task changes among all participants. It also increased the rate of completion for two of the three students: In Wal-Mart, for Brian from 1.75 to 1.83 and for Tim from 1.10 to 1.52, and in the cafeteria, for Brian from 5.25 to 7.54 and for Tim from 7.92 to 9.78. In both places, the use of the book had little effect on Hope’s rate of performance
Chang et al. [74], Taiwan	Quantitative experimental case. Observation, tape recording	N = 1, Yvonne: F, 27 years, dg: IDD	The Location-based task prompting system (Locompt) was used in a short-order snack shop. The smart phone was fastened onto the participant’s lower arm. The system was programmed to be able to generate task cues in text, sound, picture, or a combination of these. Three sets of task sets were performed by the participant. Each task set had nine task steps to carry out an order with desserts, beverages and cookies. Baseline: Task set was performed with no assistive technology Intervention phase: Task set was performed with assistive technology (Locompt)	Percentage of correct task steps	Performance During the intervention phase, the percentage of correct task steps was significantly greater (99%) than at baseline (55%). The results indicate that the Locompt system in conjunction with operant conditioning strategies may facilitate autonomous functioning of vocational jobs across multiple workstations
Devlin [75], USA	Case study. Data were collected at baseline, during the intervention phase, and during the maintenance phase	N = 4, Fred: M, 32 years, dg: moderate ID Mat: M, 20 years, dg: moderate ID, attention deficit disorder Kevin: M, 21 years, dg: mild ID, gross motor deficits Steve: M, 30 years, dg: mild cerebral palsy All worked 20 h per week and had been employed for between 2 and 4 months. They had general cleaning duties on different floors of the designated buildings	The Self-Determined Career Model was developed to enable adult service providers to help individuals become self-regulated problem-solvers, to self-direct in the career decision-making process, and to gain enhanced self-determination. The model included three phases: 1) set a career/job goal, 2) take action, and 3) assess/adjust goal or plan. The model used a generic set of questions, which represent steps in the problem-solving sequence. Questions that followed the basic framework allowed individuals to modify their own behavior and thus become self-directed in reaching their goal	Job performance (percentage of correct responses)	Job performance Fred: baseline 50–53%, during intervention session 70–80%, and during maintenance condition 77–96% Matt: baseline 34–48%, during intervention session 72–93%, and during maintenance condition 93–100% Kevin: baseline 14–36%, during intervention session 32–100%, and during maintenance condition 77–91% Steve: baseline 44%, during intervention session 64–88%, and during maintenance condition 79% The work-related performances of all four employees improved after the three phases of the Self-Determined Career Model. Positive changes were evident between baseline and intervention conditions and continued in the maintenance phase.
Dotson et al. [77], USA	Case study, observation	N = 3, Candy: F, 23 years, dg: MR Leah: F, 21 years, dg: Down syndrome Ethan: M, 27 years, dg: Down syndrome	Transition academy provided instructions for independent living and vocational skills as well as community-based opportunities to practice these skills through activities such as volunteering at a local food bank and going out to eat and shopping. Self-employment job skills were evaluated within the context of the recycling program. The Transition academy team created an analog business that the Transition Academy students were responsible for running. Participants were paid with tokens. The aim of the intervention was not to teach all the skills required to run a business without help or supervision, but to teach worker job skills (how to do the job), supervisor skills (how to supervise someone else doing the job), and office skills (how to keep records of work completed)	Job skills: Percentage of job steps performed correctly and independently	Candy performed job skills at levels well below 20% on average at baseline, improved substantially during teaching, and maintained a high level of performance of job skills during maintenance and while working shifts in the natural environment. Leah and Ethan performed job skills at lower levels at baseline, improved during teaching, and maintained a generally high level of performance of job skills during maintenance and while working shifts in the natural environment
Furniss et al. [69], UK	Quantitative case study, observation, interview of the co-workers and carers	N = 6 Mr. P: M, 35 years, dg: severe DD, job: assembling and packing nut/bolt/washer kit for classic cars Mr. W: M, 31 years, dg: severe DD, job: assembling of box and packing with forage caps Ms. R: F, 34 years, dg: severe DD, job: assembling and packing nut/bolt/washer kit for classic cars Mr. V: M, 43 years, dg: severe DD, job: preparing clock cards (attaching name sticker, sorting by dept., date stamping) Mr. H: M, 36 years, dg: severe DD, assembling aqualung pillar valve Mr. S: M, 47 years, dg: severe DD, assembling aqualung pillar valve	VICAID, palmtop-based job aid for workers with severe developmental disabilities. The system was controlled by a single large key that the worker used and a second, inconspicuous key that the supporting job coach or co-worker used. Three user prompt devices were used: a small free-standing loudspeaker unit, a smaller, portable device to which the user listened via an ear-piece, and a vibrating prompting device carried by the user on their belt or in their pocket. For each job task, a sequence of pictorial instructions created on a desktop PC was downloaded to the palmtop. The system enabled the worker to access pictorial instructions designed to help them accurately complete their tasks. It also provided reminders to access instructions, and/or alerted a job coach or supervisor if the workers had difficulty with a task	The percentage of task steps correctly completed, total time taken by the participant to complete the task, amount of time during each session that the participant spent actively engaged in the task	Job performance The computer-aided VICAID system was effective in enabling participants to perform work tasks with a high degree of accuracy following relatively brief periods of intensive training. The transition of intensive training by a job coach in the VICAID-supported maintenance condition, together with limited job coach input, led to four of the six participants showing further improvements in performance accuracy at or beyond the levels achieved with intensive training. Practice in job tasks with the computer-aided system led to both maintenance of previously learned skills and learning new job competencies
Gilson and Carter [79], USA	Quantitative case study. Single-case experimental design	N = 1 Braxton, M, 20 years, dg: ID, IQ: nr. Ethnicity: African American. Previous volunteer and employment experiences at the thrift store, restaurant, and his local church	The study was conducted the participant’s individual jobsite. Braxton worked 4 h weekly at a market within a residence hall on campus. Tasks included stocking grocery shelves, refilling supplies at the coffee station and breakfast buffet line, and marking items with price tags and expiration dates. Braxton typically had one direct supervisor, about 3 co-workers and 4–6 customers. The intervention package was comprised of a) reduced proximity, b) use of CVC (convert audio coaching, c) social-focused coaching, and d) task-related proximal coaching as needed. The participant and the coach were linked through a two-way radio with accompanying earpiece. CAC job coach was trained to focus majority of the prompts on encouraging the participant to seek assistance from others when completing a task, rather than relying on the job coach. In addition, she/he was taught to give explicit social prompts when someone was in proximity for an interaction	Percentage of intervals with task engagement and interactions.	Performance. At baseline, Braxton’s levels of social interaction were consistently low with a flat trend. He rarely initiated or responded to interactions with anyone except his job coach. After he was coached on initiating conversations with his co-workers an accelerating trend and change in level was observed (from 0.7 to 27%), and it maintained at 27% after completely removing the coach Task engagement. Braxton’s task engagement level was 98% at baseline and it slightly increased to 98 and 99% during CAC and withdrawal phases respectively
Kemp and Carr [67], USA	Quantitative, experimental case	N = 3 (2 M/1F) dg: Autism and severe mental retardation; Severe problem behavior (aggressive, self-injury) Age: 26–30 years	The intervention included three factors: a) Interventions were chosen on the basis of the hypotheses regarding the maintaining variables for problem behavior; b) the multicomponent intervention package included some combination of functional communication training, building rapport, making choices, embedding demands, and building tolerance of delay of reinforcement; c) Measures of latency of problem behavior and percentage of work steps completed were used as outcome measures. At the end of the intervention phase, the job coach trained the regular employees in the implementation of the intervention and gradually reduced their supervisory time while the regular employees increased theirs	Outcomes	Results/Outcomes: All three employees (participants) were able to complete tasks (a planting sequence in a community greenhouse) with no significant problem behavior following multicomponent intervention, and were able to work four hours at a time. In addition, the greenhouse managers reported considerable confidence in the job coach’s ability to deal with any behavior difficulties, keeping both coworkers and property safe from harm; reported that problem behavior was almost never severe following the intervention, and acknowledged the employees as productive members of the greenhouse team
McGlashing-Johnson et al. [70], USA	Quantitative case study. A multiple baseline across students was used to evaluate the effects of the intervention. Task analyses. Observations by trained observers 2–4 times per week. The students recorded their behaviors using self-monitoring cards. The Goal Attainment Scaling (GAS). Likert scale	N = 4. Jessica: F, 17 years, dg: moderate MR Sam: M, 17 years, dg: moderate MR Lindsay: F, 20 years, dg: moderate/severe MR Milo: M, 16 years, dg: moderate/severe mental retardation	All students were involved in a work-based learning program in the community, which was operated by the school district. The program involved a self-regulated problem-solving process in which students set their goals, developed and implemented action plans to enable them to achieve their goals, and evaluated their progress in achieving these goals. A job coach accompanied all students to their respective sites. Jessica: packing bread/buns for patients in the hospital, work in the dish room. Lindsay and Milo: work in the garage of a small metropolitan bus station and cleaning the interiors of the buses. Sam: work at a gardening center. He priced items, swept the floors, shoveled snow, and counted inventory	The percentage of correct responses in the task analysis for each task	Job performance The Self-Determined Learning Model of Instruction (SDLMI) represents an effective method for teaching problem-solving to people with cognitive disabilities. During baseline, after training sessions and during maintenance, the respective percentages of correct responses were for Jessica 50%, 80%, 93%, Sam 31%, 70%, 80%, Lindsay 15%, 79%, 80%, Milo 6%, 46%
McMahon et al. [78], USA	Quantitative case study. Navigation checks, interview	N = 3 Catelyn: F, 23 years, IQ: 45 Jon: M, 24 years, IQ: 56 Arya: F, 20 years, IQ: 64	The students completed a postsecondary education program. Three treatment conditions were implemented: navigation skills including a) a paper map, b) a Google Map, and c) augmented reality (AR). During each navigation session, each student was randomly assigned to one of the three treatment conditions using a spinner. This study occurred in a downtown area of a city. Participants navigated city streets to locate businesses that offered potential employment opportunities. The starting and ending localities were within a 12- to 20-minute walking distance of one another	The percentage of correct independent navigation decisions within 30 s during ‘navigation checks’ while walking to a targeted unknown business location. Responses Yes/No	Functioning/performance The AR treatment condition was the most effective. At baseline, with a paper map, a Google map, and AR application, the respective percentages of correct responses were for Catelyn 11.5%, 20.14%, 45.75, 75%, Jon 16.13%, 20.47%, 40.95%, 75%, and Arya 13.6%, 19%, 31.4%, 85.7%
Renzaglia et al. [65], USA	Quantitative case study	N = 1 Phil: M, 23 years, dg: Down syndrome, low-moderate level of functioning. Occupation: animal carer in a university vivarium (competitive employment)	A five-stage process included job task analysis, pre-baseline assessment, baseline assessment, treatment, and post-treatment follow-up. 1) Task analysis involved direct observation of co-workers performing the job tasks, performance of the tasks by the principle investigator (i.e., job coach), and reviewing the recorded task sequences with Phil’s supervisor. 2) The second state was to evaluate Phil’s current performance and to identify the specific skill areas in which training and retraining was needed. 3) The baseline data on the nine skills were collected for a five-day period. 4) Instruction in the nine skill areas identified as deficient in baseline assessment. Systematic instruction procedure using a least-to-most intrusive prompt system. 5) Post-training assessment of the subject’s performance under the same non-training conditions as those in the baseline assessment	The percentage of job tasks performed correctly in a nine-item task analysis	Job performance The percentage of correct performance in the nine designated tasks was 44% across the five days baseline, 68.7% during treatment, and 92.5% in the post-treatment assessment
Simmons and Flexer [66], USA	Quantitative case study.Observation.	N = 2 Sandy: F, 27 years, dg: moderate MR Donna: F, 30 years, dg: severe MR	The intervention was conducted in a janitorial and maintenance supported employment program at a restaurant/hotel complex. The training was carried out by a community employment specialist employed by the supported employment program to perform individualized placement and training (on Monday through Friday, 9–11 am, weekly). The training tasks were those of cleaning a restroom	The percentage of steps performed independently during baseline, training and follow-up (two months after training had ceased)	Job performance Both participants increased their performance to the criterion performance of 80%. Sandy: Performance increased from baseline (13% in Phase 1.15% in Phase 2, 54% in Phase 3) to training (81, 83, and 81%, respectively) Donna: Performance increased from baseline (46% in Phase 1, 40% in Phase 2, 54% for Phase 3) to training (89, 92, and 88%, respectively)
Taber et al. [68], USA	Case study, quantitative. Video tape, observation. Wilcoxon Matched Pairs Signed-Ranks Test	N = 5 Student 1: M, 18 years, dg: moderate MR, IQ: 41 Student 2: F, 17 years, dg: moderate MR, IQ: 43 Student 3: M, 18 years, dg: moderate MR, IQ: 42 Student 4: M, 16 years, dg: moderate MR, IQ = 41 Student 5: M, 18 years, dg: moderate MR, IQ = 40	A self-operated single-word auditory prompting system and a self-operated multiple-word (3 words) prompting system. Both systems were delivered via a tape recorder and headphones using separate, prerecorded cassette tapes with cues specific to each worker’s chain of vocational tasks in two vocational settings (church, pet store)	Number of independent task transitions across sessions in two vocational settings Performance duration	Performance A statistically significant difference was found between baseline and both auditory prompting systems in both vocational settings. The number of independent task changes ranged at baseline from 0 to 3, during intervention from 2 to 6 with the single-word and from 3 to 6 with the multiple-word prompting system. Performance duration differences between prompting systems were significant for Student 3 (T = 1.00, p < .05, N = 7) and Student 5 (T = 0.00, p < . 05, n = 7). They transitioned through tasks in significantly less time using the multiple-word auditory prompts in the pet store
Van Laarhoven et al. [80], USA	Quantitative case study. Observation	N = 2 Gerald: M, 17 years, dg: Autism and moderate ID, IQ: 44 Nick: M, 18 years, dg: Autism and moderate ID, IQ: nr	The study was conducted within the faculty conference room. The participants were responsible for cleaning and preparing the room for different meetings: configure tables and chairs, clean the white board, and throw away garbage or place important items in the lost and found. The work comprised 3 decision points. Universally-designed prompting systems presented on iPads and HP Slates were compared to improve the independent vocational performance of participants	Percentage of correct responses, percentage of media options selected by participants, the percentage of decision points correctly selected by participants across baseline and intervention phases	Performance Both participants increased their vocational skills using mobile devices. There was not a large difference in correct responding when devices were compared, but participants performed slightly better when using their preferred device. The participants selected video prompts more often than other media prompts (i.e. picture prompts) during initial sessions, except Nick during the first session. The participants effectively self-selected and self-faded their reliance on media-prompts as they became more independent with the task
West and Patton [73], USA	Quantitative case study	N = 4 Adam: M, 41 years, dg: severe ID Gena: F, 38 years, dg: ID and Rhett syndrome Alex: M, 35 years, dg: moderate ID Kylie: F, 34 years, dg: severe ID	Participants attended a community-based habilitation agency for six hours a day, Monday to Friday. The setting contained several activities including vocational tasks (i.e., sorting and assembly) and other activities such as listening to music, reading books, watching television, and kitchen and laundry tasks	The number of independent correct responses for each client during each job training session	No independent correct responses were observed for any participant during baseline sessions across task and procedures. Gena performed all five steps independently and correctly in Session 15, Kylie in session 14, Adam in Session 15, and Alex in Session 15

*N* number, *M* males, *F* females, *Dg* diagnosis, *IQ* Intellectual Quantity, *nr* not reported, *ID* Intellectual Disability, *IDD* Intellectual and Developmental Disability, *DD* Developmental Disability, *LD* Learning Disability, *MR* Mental Retardation, *SE* Supported Employment

**Table 4:** Characteristics of included qualitative case studies

Authors, year, country	Methods, study design	Population, definitions, terms	Intervention	Findings, barriers and facilitators
Aspinall [62], UK	Qualitative case study. Interview, questionnaire	N = 10 (5 M/5F) Dg: Learning disabilities Age: nr IQ: nr	The TATE project of assistive technology and telecare to improve quality of life, especially employment	Assistive technology supported the independence of people with learning disabilities
Grossi et al. [60], USA	Case study. Observation	N = 2 Dana: F, 28 years, dg: mild ID, IQ: 64 Rick, M, 28 years, dg: borderline, IQ: 70	The workplace was a restaurant. The aim was to enhance social interaction. The social interaction situations were tape-recorded prior to and during the work shifts. The recordings were analyzed together with the participants, and during these analysis sessions the participants received guidance in social interaction skills	Prior to the intervention Dana did not pay attention or reacted negatively to her supervisor and workmates; she also had difficulties accepting critique Rick was very sensitive to critique. He tried to avoid other people and also behaved obsessively and was paranoid After the intervention Both Dana and Rick were more responsive to the initiatives of the others and in a socially acceptable manner
Hagner and Davies [53], USA	Case-study, interviews of entrepreneurs and support people	N = 7 Rhonda: F, age: nr, dg: ID Pat: F, age: nr, dg: ID, physical disability. Jenny: F, age: nr, dg: ID, physical disability Paul: M, age: nr, dg: ID Maxwell: M, age: nr, dg: ID Peggy: F, age: nr, dg: ID Richard: M, age: nr, dg: ID, physical disability	People with ID as entrepreneurs. Entrepreneurship was built according to each person’s own interests and values. Rhonda, Pat, Jenny and Maxwell received business training. The established firms had no personnel other than the entrepreneur. Each entrepreneur had their own support person, who took responsibility for the firm’s practical issues	Factors enhancing employment: Only a small monetary investment was needed to start the business. Most of these people only worked part-time. Some of them had the support person with them all the time, others only occasionally. Work as an entrepreneur was flexible, independent, and they could do work that was interesting Barriers to employment: The income from the business was low, so other sources of income were needed. The support people did not have enough expertise or interest in business. It was difficult to obtain support for running a business, and to make social contacts
Ham et al. [64], USA	Case study	N = 1 Kristen: F, age: nr (young adult), dg: Down syndrome, autism, and hearing disability	Kristen worked in the hospital delivery ward. Her jobs were cleaning the babies’ nursing room, cleaning computers, phones and kitchens, and taking care of blankets and clothes in the nursery. The aim of the positive behavior program was to decrease her disruptive behavior and to help her better complete her duties. The program included self-assessment, a timetable in pictorial form and auditive reminders. The program was planned by a positive behavior support person and her workmates took part in its implementation	Prior to the intervention: Kristen behaved disruptively at the workplace, and was unable to carry out her duties. She was in danger of being dismissed After the intervention: Kristen was more self-regulative and independent. In three months her refusals to comply with the timetables decreased by 79%, refusals to follow instructions decreased by 59% and refusals to handle the alarms decreased by 67%. Her workmates felt it was more comfortable to work with her. Kristen continued to work in the hospital for two more years. After that she moved to another city and obtained another job
Jarhag et al. [63]	Case study	N = 2 Anna: F, 30 years, dg: ID (50% disability allowance), IQ: nr, had attended to a school for children with ID Adam: M, 21 years, dg: ID, IQ: nr, had attended to a school for children with ID	Model for special support and follow-up. The support person helped the employee plan work tasks and gave individual support to the person with ID according to the plan. The support person was present at the workplace and supervised the tasks, and also helped the employee modify working conditions. The amount of support given by the support person gradually diminished, and when the employee succeeded in their tasks independently, the support ended. The maximum duration of support was six months	Prior to intervention: Anna worked as a wage-subsidized cleaner for two years. When she moved to another city the wage-subsidy ended. A year later, after her maternity leave, Anna enrolled as a job-seeker, and temporarily attended a sheltered workshop until a job was found for her. However, she did not find employment. The employees felt that Anna could not be employed because of her shyness, lack of initiative and low stress tolerance Adam was registered as a job-seeker in the employment office After the intervention Anna was employed with a wage subsidy in the hotel and restaurant industry. Her job tasks were tailored according to her skills. Later Anna returned to being a customer of the employment office. The support person helped her and again she obtained a wage-subsided job Adam: The support person helped him while he was in his last year of school for people with ID. After school, Adam worked as a trainee, but this did not lead to employment. The support person arranged another job for him, but this did not last very long. Following discussions with the support person, Adam again obtained a place as a trainee, which later turned into a wage-subsided post The factors enabling employment were sufficient duration of support, individually tailored tasks and wage subsidies
Wehman et al. [61], USA	Qualitative case study, observations	N = 2. Karen, F, 18 years, dg: severe ID, autism, communication deficits, IQ: nr, at school Lisa, F, 22 years, dg: moderate/severe ID, IQ: nr	Employment with support of family, job coach and workmates. The employees’ performance was evaluated at home and in job tasks, and on the grounds of these evaluations, suitable job tasks and workplaces were defined. The intervention included choosing the workplace and the employee, getting to know these; and guidance, training and support at the workplace	Both Karen and Lisa obtained employment. Karen worked at a restaurant, where her tasks where first spreading flour and later also handling dirty dishes. Lisa’s first workplace was at a café at school, and after that a grocer’s, where she had simple tasks such as towel folding Factors promoting employment and work performance: support from family and job coach, support and opportunities to develop at work

*N* number, *M* males, *F* females, *Dg* diagnosis, *IQ* Intellectual Quantity, *nr* not reported, *ID* Intellectual Disability, *IDD* Intellectual and Developmental Disability, *DD* Developmental Disability, *LD* Learning Disability, *MR* Mental Retardation, *SE* Supported Employment

#### Participants

The total number of participants in the 38 studies was 2,41,080. The quantitative studies included 35–1,21,335 participants (n = 2,39,506), the qualitative studies 3–27 participants (n = 58), the mixed method study included 1452 participants and the case studies 1–10 participants (n = 64).

The age of the participants varied between 16 and 51. However, two qualitative studies [53, 54] and two case studies [62, 64] did not report the age of the participants. The proportion of men and women differed in the studies. In the quantitative studies, the proportion of women varied from 29% [50] to 58% [47]. In one qualitative study [56] all the participants were men and in another qualitative study [54] all the participants were women. Two studies [51, 59] did not report the gender of the participants. Seven studies reported the ethnicity of the participants [45, 47–49, 51, 52, 79].

Sixteen of the 38 studies reported the degree (mild, moderate, severe, profound) of ID as a background factor of the participants. People with mild ID participated in two cohort studies [48, 51], one qualitative study [57], and one case study [60]. People with either mild or moderate ID participated in three case studies [71, 75, 76], and people with moderate ID participated in two case studies [68, 80]. Renzaglia et al. [65], Simmons and Flexer [66], McGlashing-Johnson et al. [70], and West and Patton [73] focused on people with moderate or severe disabilities. People with severe ID were participants in three case studies [61, 67, 69]. Two studies [45, 77] reported the participants ‘level of functional capacity.

Seven of 38 studies concerned students [46, 48–52, 59], one study [47] concerned people who worked in sheltered workshops, and one study [44] concerned job-seekers. Four studies reported the occupation or work tasks of the participants as the background factors [69, 72, 75, 79].

#### Interventions

All studies contained various mixtures of intervention components. The interventions were carried out during secondary education, the transition from education to work, job-seeking and sheltered work. In general, the content of the intervention was briefly described. However, the stages of the intervention process were poorly reported, and the term rehabilitation was only seldom used.

During secondary education (Upper Secondary School for Pupils with ID) (ICF code d825), the students participated in different educational programs such as national programs, specially designed programs, vocational training and training activities or graduated with inadequate grades [50]. National programs focused on different parts of the labor market, for example, vehicles and transportation, hotels and restaurants, or social and healthcare. Some specially designed programs or individually tailored education were also on offer. The educational programs aimed to improve vocational qualifications and work awareness. The vocational qualification courses included assisting smaller pupils during classes, helping the janitor and working in the school kitchen. Work awareness training courses included watching videos about work, talking about presenting oneself at work, and health and safety instructions at school [46].

Some interventions included postschool and transition services (ICF code e5900) during education to improve the transition from education to work [43, 48, 49, 51, 55, 75]. Postschool services included postsecondary education institution accommodations and services, job training services, and life skills services [51]. Employment-related transition services were, for example, vocational assessment, career counseling, pre-vocational education, career-related technical or vocational education, pre-vocational or job readiness training, instructions for job-seeking, job shadowing, job coaching, special job skills training, placement support, internship or apprenticeship programs, work experience at school and other paid work experience [44, 48, 55, 65]. Job tasting was a short, unpaid, time-limited work experience period at the workplace which allowed people to sample a variety of different jobs and work cultures [44]. One qualitative study [57] analyzed the different actors’ experiences (schools, agencies, parents, other people) of the transition process and of employment (e310), and Fasching’s [54] study analyzed the experiences of people with ID of the transition from school to vocational training and employment.

The interventions also included supported work (supported employment, SE) (d855) [66], the use of a job coach or other support person (e340, e360) [45, 63], and designing personal solutions (e5900) [64]. One cohort study [46] analyzed whether attending a sheltered workshop (e5900) improved the employment outcomes of supported employees with ID.

Some interventions developed the independence of people with ID, applying the ICF model to the areas of body structures/functions, activities and participation. The interventions included support in psychological and behavioral issues (behavior) (b122) [56, 64] and communication abilities (d3) [67]; and a self-regulated problem-solving process in which students set their goals, developed and implemented their action plans (b164, d175) [70], made career-related decisions (d177) [75], were able to perform daily activities (d620, d630) [77], learned navigation skills such as finding the workplace and using public transportation (d4602) [78], and gradually reduced help in carrying out work tasks (d850) [72, 75, 77].

The use of digital solutions (e135) was intended to improve the daily work performance of people with ID in the open labor market. They could use these tools for receiving digital instructions for work tasks, work processes, work techniques or schedules. These solutions consisted of video modeling and audio coaching [72, 76], photo activity schedule books [71], smart phones [74], palmtop-based job aids [69], and self-operated auditory prompting systems [68, 80]. In the case studies, the interventions were carried out at workplaces such as restaurants [60, 66], hospitals [64], factories and warehouses [76], at markets [79], at schools [70, 72], and in conference rooms [80].

#### Outcomes

The outcomes were employment in the open labor market [43, 45, 47, 51, 52], transition from school to the open labor market [44, 46–48, 54, 55], and work performance [43, 44]. The outcomes in all 16 quantitative experiments were job skills and work performance [65–80].

#### Study quality

The RCT study [43] was considered to be of high methodological quality, with scores of 11 out of 22, and the CCT study of Kilsby and Beyer [44] was of low methodological quality, with scores of 9 out of 22 according to van Tulder et al. [32]. The methodological quality of four cohort studies [47, 49, 50, 52] out of six were high, with the maximum of nine ‘stars’ according to Wells et al. [36]. Four cohort studies [45, 46, 48, 51] and one multimethod study [59] were considered to be of intermediate quality. All six qualitative studies [53–58] were considered to be of high quality, with scores ranging from 12 to 20 out of 20, according to the modified CASP method. (Table 6–9, Supplementary Files).

#### Effectiveness

The quantitative studies showed that supported work increases the employment of people with ID in the open labor market (Table 5). This result was based on one high-quality RCT study [43], one high-quality cohort study [47], and one moderate-quality cohort study [45] of altogether 16,947 people with ID. The quantitative studies also showed that both secondary and postsecondary education, including support services and work training, increased the transition of people with ID from school to the open labor market. The result was based on two high-quality cohort studies [50, 52] and three moderate-quality cohort studies [46, 48, 51] with 2,07,484 participants altogether.

**Table 5:** Summary of results

Outcome	Percentage and/or comparative risk (95% Confidence Interval)		Number of subjects	Evidence (GRADE)	Study
	Intervention	Control group			
Employment in open labor market	Supported work 21%	Sheltered employment 0%	49	Moderate	High quality RCT study [43]
	Employment to supported work after sheltered work 59.6%	Employment to supported work without sheltered work 60.4%	9,808	Low	High quality cohort study [47]
	Employment after postsecondary education (OR 2.0)	Employment without postsecondary education	11,280	Low	High quality cohort study [52]
	Support of work coach 6.1%	No control group	7,090	Very low	Moderate quality cohort study [45]
Transfer from school to open labor market	Support services and work training during secondary education 22.4%	No control group	12,269	Low	High quality cohort study [50]
	Support activities during education 75.9% One transition activity during education (OR 1.2) Work period during education (OR 3.5) Work experience during education (OR 5.7)	No control group	62,513	Very low	Moderate quality cohort study [48]
	Work experience and training of work awareness 21%, (OR 1.01)	No control group	87	Very low	Moderate quality cohort study [46]
	Work briefing and work training during secondary education 45%/2008, 11%/2009	No work training or work experience during education 6%/2008, 5%/2009	1452	Very low	Moderate quality multimethod study [59]
	Personal educational program and transition services at age of 14 years 74.3%	Personal educational program and transition services at age of 16 years 57.8%	15,040	Low	High quality cohort study [49]
Work performance	Supported work Before intervention: 54 (SD 6) After intervention: 51 (SD 8)	Sheltered work Before intervention: 53 (SD 8) After intervention: 54 (SD 7)	49	Moderate	High quality RCT study [43]
	Short work tastings, self-evaluation, education of job coaches. Need for job coaches decreased: at beginning 4.79 > 4.04 or 2.80.	No control group	35	Very low	Moderate intervention study [44]

However, on the basis of one high-quality RCT study [43] and one high-quality cohort study [47] covering a total of 15,089 participants, sheltered work did not increase the employment of people with ID in the open labor market (Table 5).

#### Barriers to and facilitators of employment

The qualitative studies concerned both the barriers to and the facilitators of employment in the open labor market (Table 2). The main barriers to employment were that the school and service system tried to guide people with ID towards traditional services such as sheltered work, sometimes against their own needs and interests [57]. However, these people’s work skills were not developed or their needs for support were not noticed in sheltered work [58]. Further barriers were discrimination in vocational experience after leaving school [54], poor experiences of class teaching and lack of work experience [58, 59].

The main facilitators of employment were people’s own activity and support from their families (e310) [57], effective job coaching (e360) [58], a well-designed work environment (e2) [61], appreciation of their work and support from their employer and work organization [56]. Other facilitators were the knowledge and experience of work during education [59], and for entrepreneurs, the use of a support person [53].

#### Work performance

The case studies showed that the use of digital solutions (e135) improved the work performance of people with ID in the open labor market. Work performance was measured as the percentage and rate of the tasks completed correctly [69, 72], the number of movements measured by a costume [76], the number of independent task changes [71], task steps [74], and percentage of intervals with task engagement and interactions [79].

## Discussion

People with ID still face specific difficulties in gaining employment, which leads to inequality and social exclusion. Personally-tailored support services, occupational education, work experience and digital solutions can be used to help these individuals become employed in the open labor market.

This systematic review covered 38 studies (one RCT, one CCT, eight cohort studies, six qualitative studies, one multimethod study, and 21 case studies) that investigated the effectiveness of, barriers to or facilitators of employment among people with ID. The main outcome was employment in the open labor market through either supported or non-supported work, transition from education or from sheltered work to the open labor market, or functioning at work.

The quantitative studies showed that secondary and postsecondary education increases the employment of people with ID in the open labor market when it includes work experience and personal support services. Supported employment also increases employment in the open labor market, but sheltered work does not. The results were in line with those of earlier studies [2, 22, 81] which have shown the support of a job coach to be important for people with ID to find work, start work and continue at work among.

The qualitative studies showed the barriers to and facilitators of employment in the open labor market among people with ID. The barriers to employment were that the school and service system tried to guide people with ID towards traditional services such as sheltered work; that these people were discriminated against at school and had negative experiences of class teaching; and that they did not obtain work experience during education. These results support the findings of earlier studies [6, 22] which have shown low rates of employment when individuals come from sheltered work to the open labor market.

The facilitators of employment were the people’s own activity and support from their families, job coaches, work environments with necessary accommodations, appreciation of their work, support from an employer and work organization, knowledge and experience of work during education; and for entrepreneurs, the use of a support person. These findings support earlier results that have shown the importance of job coaches, employers’ responsibility [82] and personally designed support services [17, 18, 20].

The case studies showed that the work performance of people with ID could be improved through the use of digital solutions in daily work. However, digital solutions are only rarely used among people with ID. One reason may be the employees’ low competence in using digital systems, especially in sheltered work. Services in general are becoming increasingly digital, and this also requires more competence from people with ID [83].

### Methodological discussion of included studies

Better reporting of basic methodological quality issues is generally required. A better description of participants, such as their gender, age, education, occupation and work experience is also needed when they are people with ID. We can conclude that people with ID are not seen as professionals because, although the studies described their diagnosis or disability well, they did not emphasize their competence and strengths, educational background or work experience. This is also true of studies of disability groups [35].

The studies included several different concepts of disability, rehabilitation, education, performance, and service systems. The concept of ID was defined as ‘developmental disability’, ‘mental retardation’, and ‘mental disability’. In the UK, ‘learning disability’ is used in the same way as ID, but in other countries, ‘learning disability’ means difficulties in learning without ID.

The articles failed to clearly report some elements of the interventions. They should have reported more information about the process and implementation schedule of rehabilitation, the initiator and the place. These shortfalls were found especially in cohort studies that focused on results and background factors such as the participants’ diagnoses.

Only few randomized controlled interventions have been carried out to enhance the employment among people with ID. This is possibly because of the low employment rate of people with ID, the low number of implemented rehabilitation programs, the ethical aspects of the study designs, negative attitudes, and a lack of financing instruments for such studies.

The outcomes in this review were employment in the open labor market in either supported or non-supported work, the transition from education or from sheltered work to the open labor market, or functioning at work. With the ICF model as a framework, these outcomes belong to ‘participation’. According to the ICF model, rehabilitation belongs to the ‘environmental factors’ that affect ‘activity’ (e.g., functioning) as do most of the synthesized themes from the analysis of qualitative studies. Obviously, the primary aim of rehabilitation on a personal level is to enhance the functioning and work ability of people with ID and to enable them to work in the open labor market. On the societal level, it is important to develop and implement solutions that enhance employment and participation and are simultaneously cost effective [17].

### Strengths and limitations of this review

The strengths of this review included its multi-scientific research group, the comprehensiveness of the searches, the use of the ICF model as the theoretical framework, and the inclusion of a wide range of studies. The reviewers were experts in different scientific areas, including both quantitative and qualitative methodology and systematic reviews. Every effort was made to insure a comprehensive search. It is possible, however, that we did not find all the relevant studies. Another limitation was that the included studies used different concepts of ID. We included quantitative, qualitative, multimethod and case studies, which showed different kinds of knowledge regarding the process and the effectiveness of rehabilitation. However, this was also a shortcoming of this study because a meta-analyses of several types of results was complicated.

### Quality assessment

This systematic review was of 38 studies (one RCT, one CCT, eight cohort studies, six qualitative studies, one multimethod study, and 21 case studies). The quality of the RCT study was assessed using the validated method of van Tulder et al. [32], which has also been used in several other reviews. The quality of the CCT study was low, mainly due to the fact that the study design did not include randomization, treatment allocation, blinding, or intention to treat the analysis. The quality of the cohort studies and the mixed method study were assessed using the Newcastle–Ottawa Scale [36]. The validity and reliability of this method was only partly evaluated in that the content validity and inter-rater reliability were established, but the criterion validity and intra-rater reliability were still in progress [36].

The original qualitative assessment tool CASP was not perceived as very powerful in differentiating between high- and low-quality studies. It only measured whether certain basic items that are essential identifiers of high-quality research were mentioned in the report. This type of measurement is crude and makes the scale difficult to use when some of the criteria are implicit in the study. Furthermore, a ‘Yes or No’ scale does not capture the fact that certain items in the CASP criteria may be more crucial to the quality of the study than others. Adding a third level of assessment, ‘Partially’, to the method, may solve the first problem. However, the second problem remains: Of the three problematic points of the qualitative studies evaluated, researcher effect is a self-evident fact connected to any study of social life, and is thus less informative than reporting the contribution of a particular study to existing knowledge. Although CASP offers a good basis for evaluating qualitative research reports, it can be further developed by giving different weights to different criteria.

The comparison of the results of the assessment of the quantitative and qualitative studies revealed a bias in that the quality assessment of qualitative studies resulted in several high-quality studies, whereas the quantitative assessment yielded only a few. This was, of course, partly due to different evaluation methods, but it may also be an indication of the different nature of these two types of research. Qualitative studies report interesting new observations about the ways in which participants observe, understand, or experience the phenomenon studied, while quantitative studies aim to make generalizations about possible causes and effects, and reveal other connections between the variables describing the phenomenon being studied. As the knowledge gained through qualitative research is descriptive and not numerical by nature, ranking studies is also difficult.

## Conclusions

More people with ID could be employed through personally tailored services and measures. Tailoring can mean secondary or postsecondary education, including proper teaching methods and personal support services, the use of supported work, workplace accommodations, and the support of one’s family and employer. Our results can be utilized in the development of rehabilitation, education and employment of people with ID, to provide them with opportunities to work in the open labor market and to participate in society.

## Electronic supplementary material

Below is the link to the electronic supplementary material.

Supplementary material 1 (DOCX 88 kb)

Supplementary material 2 (DOCX 26 kb)
