# Supplementary material for: The Effectiveness of Rehabilitation Interventions on the Employment and Functioning of People with Intellectual Disabilities: A Systematic Review
Source: J Occup Rehabil. 2019 May 16;29(4):773–802. doi: 10.1007/s10926-019-09837-2 (PMC6838041; doi:10.1007/s10926-019-09837-2)
Supplement: Supplementary file 1 — Supplementary material 1 (DOCX 88 kb) [file 10926_2019_9837_MOESM1_ESM.docx]

# Literature search for systematic review update September 2016 *Rehabilitation among people with intellectual disabilities: A systematic review of its effectiveness on their functioning and participation in work life, and of the barriers to and facilitators of employment.* Information specialist: Pia Pörtfors, National Institute for Health and Welfare

**Limits:** September 2016 – February 2019

Aika 5.2.2019 14-16, 16.30-19.30
6.2. 10-14
7.2. 10-11, 15-19

**Databases included:**
Cinahl (EBSCO), Cochrane Central Register of Controlled Clinical Trials , Cochrane Database of Systematic Reviews, EMBASE (OVID), ERIC (ProQuest), Medline (OVID), Medic, OTseeker, PEDro, PsycInfo (EBSCO), Pubmed, SocIndex with fulltext (EBSCO), Web of Science
 **Other data sources included:**Google Scholar, BASE (Bielefield Academic Search Engine)

**Number of records** **per database or data source, number of duplicates**
Databases and datasources appear in the order they were searched.

| **Database/data source** | **No of records Update search** |
| --- | --- |
| Medline (OVID) | 51 |
| Cinahl (EBSCO) | 56 |
| Web of Science | 14 |
| PsycInfo (EBSCO) | 28 |
| Cochrane Central Register of Controlled Clinical Trials | 3 |
| Cochrane Database of Systematic Reviews | 0 |
| SocIndex with fulltext | 10 |
| EMBASE | 122 |
| OTseeker | 0 |
| PEDro | 1 |
| Medic | 3 |
| ERIC (ProQuest) | 20 |
| PubMed | 24 |
| BASE (Bielefield Academic Search Engine) | 118 |
| Google Scholar | 26 |
| Number of records: | 476 |
| Number of records after duplicate removal: | 277 |

**Search strategies**

**Epub Ahead of Print, In-Process & Other Non-Indexed Citations, Ovid MEDLINE(R) Daily and Ovid MEDLINE(R) 5.2.2019**

1 exp Intellectual Disability/ OR Mentally Disabled Persons/ OR Developmental Disabilities/ (110215)

2 ("Intellectual* disab*" OR "intellectual development Disorder*" OR "intellectual and developmental dis*" OR "developmental* disab*" OR "mental* retard*" OR "mental* handicap*" OR "learning disab*" OR Down's OR "Down Syndrome").ti,ab,kf. (63793)

3 OR/1-2 (134602)

4 exp Rehabilitation/ OR Rehabilitation, Vocational/ OR Vocational education/ OR Education of intellectually disabled/ OR Remedial Teaching/ OR Education, Special/ OR Sheltered Workshops/ OR exp Self-help Devices/ OR Self Care/ OR Intervention Studies/ OR "Mainstreaming (Education)"/ OR "Early Intervention (Education)"/ OR exp Therapeutics/ OR Vocational Guidance/ OR Socialization/ OR Social Support/ OR Behavior Therapy/ OR Person-Centred Planning/ (4392609)

5 (rehabilitat* OR treatment* OR habilitation OR therap* OR training OR education* OR program* OR "assistive technolog*" OR "assistive devic*" OR "self-help devic*" OR intervention* OR "career counseling" OR "social support" OR "self management support" OR "self care").ti,ab,kw. (7060735)

6 (rh OR th).fs. (1917136)

7 OR/4-6 (93854384)

8 exp Employment, Supported/ OR exp Employment/ OR exp Unemployment/ OR Work Capacity Evaluation/ OR Workplace/ OR Sheltered Workshops/ (86152)

9 ("sheltered employment" OR "sheltered Work" OR employment OR employab* OR "Work ability" OR "Work capacity" OR "ability to Work" OR "vocational status" OR "vocational adjustment").ti,ab,kw. (59241)

10 (("transition to" OR "entry into" OR "entry to" OR entering) adj3 (Work OR labo?r OR vocation*)).ti,ab,kw. (1146)

11 OR/8-10 (128414)

12 exp Clinical Trial/ OR Case-control Studies/ OR exp Cohort Studies/ OR Controlled Before-After Studies/ OR Review/ OR Comparative effectiveness research/ OR Qualitative research/ OR Quantitative Evaluation/ OR Evaluation Studies/ OR Validation Studies/ OR Program Evaluation/ OR Follow-Up Studies/ OR Longitudinal Studies/ OR Concurrent Study/ OR Prospective Study/ OR Treatment Outcome/ OR Patient Outcome Assessment/ (5771030)

13 (randomized controlled trial OR controlled clinical trial OR clinical trial OR comparative study OR evaluation studies OR case reports OR meta-analysis).pt. (4584907)

14 (CCT OR RCT OR random* OR "clinical trial*" OR "clinical study" OR "controlled clinical" OR "controlled trial*" OR "controlled study" OR "case-control*" OR Cohort OR "prospective study" OR "concurrent study" OR "follow up" OR longitudinal* OR "comparative study" OR "before-after" OR "case stud*" OR "systematic review*" OR meta-analysis OR "qualitative study" OR "qualitative design" OR "qualitative method*" OR "quantitative study" OR "quantitative design*" OR "quantitative method*" OR "validation study" OR "evaluation study" OR "effectiveness study" OR "program evaluation" OR "program* effectiveness" OR "treatment outcome*" OR "outcome* assessment").ti,ab,kw. (3039045)

15 OR/12-14 (9692655)

16 3 and 7 and 11 and 15 (450)

17 limit 16 to ed=20160906-20190205(51)

**Cinahl 5.2.2019**

S10 S8 AND S9 (56)

S9 DT 20160906-20190205 (981,965)

S8 S1 AND S2 AND S3 AND S7 (293)

S7 S4 OR S5 OR S6 (1,568,876)

S6 TI (CCT OR RCT OR random* OR "clinical trial*" OR "clinical study" OR "controlled clinical" OR "controlled trial*" OR "controlled study" OR "case-control*" OR Cohort OR "prospective study" OR "concurrent study" OR "follow up" OR longitudinal* OR "comparative study" OR "before-after" OR "case stud*" OR "systematic review*" OR meta-analysis OR "qualitative study" OR "qualitative design" OR "qualitative method*" OR "quantitative study" OR "quantitative design*" OR "quantitative method*" OR "validation study" OR "evaluation study" OR "effectiveness study" OR "program evaluation" OR "program* effectiveness" OR "treatment outcome*" OR "outcome* assessment") (310,133)

S5 AB (CCT OR RCT OR random* OR "clinical trial*" OR "clinical study" OR "controlled clinical" OR "controlled trial*" OR "controlled study" OR "case-control*" OR Cohort OR "prospective study" OR "concurrent study" OR "follow up" OR longitudinal* OR "comparative study" OR "before-after" OR "case stud*" OR "systematic review*" OR meta-analysis OR "qualitative study" OR "qualitative design" OR "qualitative method*" OR "quantitative study" OR "quantitative design*" OR "quantitative method*" OR "validation study" OR "evaluation study" OR "effectiveness study" OR "program evaluation" OR "program* effectiveness" OR "treatment outcome*" OR "outcome* assessment") (694,575)

S4 MH("Outcomes (Health Care)+" OR "Outcome Assessment" OR "Treatment Outcomes+" OR "Outcomes of Education" OR "Randomized Controlled Trials" OR "Controlled Before-After Studies" OR "Case Control Studies+" OR "Clinical Trials+" OR "Prospective Studies+" OR "Concurrent Prospective Studies" OR "Nonconcurrent Prospective Studies" OR "Program Evaluation" OR "Postexposure Follow-Up" OR "After Care" OR "Qualitative Studies+" OR "Phenomenology" OR "Multimethod Studies" OR "Grounded Theory" OR "Field Studies" OR "Quantitative Studies" OR "Meta Analysis" OR "Validation Studies" OR "Clinical Effectiveness" OR "Cost Benefit Analysis" OR "Comparative Studies" OR "Summative Evaluation Research" OR "Formative Evaluation Research" OR "Work Capacity Evaluation" OR "Evaluation Research+") (1220,262)

S3 ( TI ( (("transition to" OR "entry into" OR "entry to" OR entering) N2 (Work OR labo?r OR vocation*)) ) OR AB ( (("transition to" OR "entry into" OR "entry to" OR entering) N3 (Work OR labo?r OR vocation*)) ) ) OR ( TI ( ("sheltered employment" OR "sheltered Work" OR employment OR employab* OR "Work ability" OR "Work capacity" OR "ability to Work" OR "vocational status" OR "vocational adjustment") ) OR AB ( ("sheltered employment" OR "sheltered Work" OR employment OR employab* OR "Work ability" OR "Work capacity" OR "ability to Work" OR "vocational status" OR "vocational adjustment") ) ) OR ( (MH "Employment+") OR (MH "Employment of Disabled+") OR (MH "Self Employment") OR (MH "Temporary Employment") OR (MH "Part Time Employment") OR (MH "Employment, Supported") OR (MH "Employment Status") OR (MH "Occupations and Professions+") OR (MH "Sheltered Workshops") OR (MH "Work Capacity Evaluation") OR (MH "Job Interviews+") ) (135,061)

S2 ( TI ( (rehabilitat* OR treatment* OR habilitation OR therap* OR training OR education* OR program* OR "assistive technolog*" OR "assistive devic*" OR "self-help devic*" OR intervention* OR "career counseling" OR "social support" OR "self management support" OR "self care") ) AND AB ( (rehabilitat* OR treatment* OR habilitation OR therap* OR training OR education* OR program* OR "assistive technolog*" OR "assistive devic*" OR "self-help devic*" OR intervention* OR "career counseling" OR "social support" OR "self management support" OR "self care") ) ) OR ( (MH "Rehabilitation+") OR (MH "Rehabilitation, Psychosocial+") OR (MH "Rehabilitation Centers+") OR (MH "Rehabilitation, Cognitive") OR (MH "Rehabilitation, Community-Based") OR (MH "Rehabilitation, Vocational+") OR (MH "Rehabilitation Nursing") OR (MH "Assistive Technology Services") OR (MH "Vocational Education") OR (MH "Remedial Teaching") OR (MH "Medication Treatment") OR (MH "Medication Care+") OR (MH "Schools, Special") OR (MH "Education, Special+") OR (MH "Mainstreaming (Education)") OR (MH "Individuals with Disabilities Education Act") OR (MH "Vocational Guidance") OR (MH "Teaching, Guidance, and Counseling (Omaha)") OR (MH "Support, Psychosocial") OR (MH "Socialization+") OR (MH "Early Intervention") OR (MH "Early Childhood Intervention") OR (MH "Behavior Therapy+") OR (MH "Cognitive Therapy+") OR (MH "Behavior Modification+") OR (MH "Transitional Programs+") ) (641,542)

S1 ( TI ( ("Intellectual* disab*" OR "intellectual development Disorder*" OR "intellectual and developmental dis*" OR "developmental* disab*" OR "mental* retard*" OR "mental* handicap*" OR "learning disab*" OR Down's OR "Down Syndrome") ) OR AB ( ("Intellectual* disab*" OR "intellectual development Disorder*" OR "intellectual and developmental dis*" OR "developmental* disab*" OR "mental* retard*" OR "mental* handicap*" OR "learning disab*" OR Down's OR "Down Syndrome") ) ) OR ( (MH "Developmental Disabilities") OR (MH "Intellectual Disability") OR (MH "Down Syndrome") OR (MH "Mentally Disabled Persons") OR (MH "Mental Retardation, X-Linked+") ) (63,586)

**Web of Science 5.2.2019**

TI=("Intellectual* disab*" OR "intellectual development Disorder*" OR "intellectual and developmental dis*" OR "developmental* disab*" OR "mental* retard*" OR "mental* handicap*" OR "learning disab*" OR Down's OR "Down Syndrome") AND TI=(rehabilitat* OR treatment* OR habilitation OR therap* OR training OR education* OR program* OR "assistive technolog*" OR "remedial teaching" OR mainstreaming OR vocational OR "assistive devic*" OR "self-help devic*" OR intervention* OR therap* OR "person-centred planning" OR "career counseling" OR "social support" OR "self management support" OR "self care") AND TI=("sheltered employment" OR "sheltered Work" OR employment OR employab* OR "Work ability" OR "Work capacity" OR "ability to Work" OR "vocational status" OR "vocational adjustment") Timespan: 2016-2019. Indexes: SCI-EXPANDED, SSCI, A&HCI, ESCI (14)

**PsycInfo 29.3.2016 5.2.2019**

S14 S3 AND S6 AND S10 AND S13 (28)
Limiters - Published Date: 20160901-20190231

S13 S11 OR S12 (743,661)

S12 TI ( (CCT OR RCT OR random* OR "clinical trial*" OR "clinical study" OR "controlled clinical" OR "controlled trial*" OR "controlled study" OR "case-control*" OR Cohort OR "prospective study" OR "concurrent study" OR "follow up" OR longitudinal* OR "comparative study" OR "before-after" OR "case stud*" OR "systematic review*" OR meta-analysis OR "qualitative study" OR "qualitative design" OR "qualitative method*" OR "quantitative study" OR "quantitative design*" OR "quantitative method*" OR "validation study" OR "evaluation study" OR "effectiveness study" OR "program evaluation" OR "program* effectiveness" OR "treatment outcome*" OR "outcome* assessment") ) OR AB ( (CCT OR RCT OR random* OR "clinical trial*" OR "clinical study" OR "controlled clinical" OR "controlled trial*" OR "controlled study" OR "case-control*" OR Cohort OR "prospective study" OR "concurrent study" OR "follow up" OR longitudinal* OR "comparative study" OR "before-after" OR "case stud*" OR "systematic review*" OR meta-analysis OR "qualitative study" OR "qualitative design" OR "qualitative method*" OR "quantitative study" OR "quantitative design*" OR "quantitative method*" OR "validation study" OR "evaluation study" OR "effectiveness study" OR "program evaluation" OR "program* effectiveness" OR "treatment outcome*" OR "outcome* assessment") )  (636,766)

S11 (((((((((DE "Between Groups Design" OR DE "Meta Analysis" OR DE "Random Sampling" OR DE "Experiment Controls" OR DE "Clinical Trials" OR DE "Treatment Effectiveness Evaluation" OR DE "Qualitative Research" OR DE "Quantitative Methods" OR DE "Treatment Outcomes" OR DE "Treatment Effectiveness Evaluation" OR DE "Mental Health Program Evaluation" OR DE "Educational Program Evaluation" OR DE "Course Evaluation" OR DE "Vocational Evaluation" OR DE "Program Evaluation" OR DE "Prospective Studies" OR DE "Longitudinal Studies" OR DE "Experimental Design" OR DE "Quasi Experimental Methods" OR DE "Cohort Analysis" OR DE "Followup Studies" OR DE "Retrospective Studies" )) (228,771)

S10 S7 OR S8 OR S9 (79,261)

S9 TI ( (("transition to" OR "entry into" OR "entry to" OR entering) N2 (Work OR labo?r OR vocation*)) ) OR AB ( (("transition to" OR "entry into" OR "entry to" OR entering) N3 (Work OR labo?r OR vocation*)) ) (2,029)

S8 TI ( ("sheltered employment" OR "sheltered Work" OR employment OR employab* OR "Work ability" OR "Work capacity" OR "ability to Work" OR "vocational status" OR "vocational adjustment") ) OR AB ( ("sheltered employment" OR "sheltered Work" OR employment OR employab* OR "Work ability" OR "Work capacity" OR "ability to Work" OR "vocational status" OR "vocational adjustment") ) (51,011)

S7 (DE "Employment Status" OR DE "Self-Employment" OR DE "Supported Employment" OR DE "Employability" OR DE "Vocational Maturity" OR DE "Vocational Evaluation" OR "Job Performance" OR "School to Work transition")  (41,800)

S6 S4 OR S5 (702,027)

S5 TI( (rehabilitat* OR treatment* OR habilitation OR therap* OR training OR education* OR program* OR "assistive technolog*" OR "assistive devic*" OR "self-help devic*" OR intervention* OR "career counseling" OR "social support" OR "self management support" OR "self care") ) AND AB( (rehabilitat* OR treatment* OR habilitation OR therap* OR training OR education* OR program* OR "assistive technolog*" OR "assistive devic*" OR "self-help devic*" OR intervention* OR "career counseling" OR "social support" OR "self management support" OR "self care") ) (462,717)

S4 ((((((DE Intervention OR DE "Rehabilitation Education" OR DE "Vocational Rehabilitation" OR DE "Vocational Education" OR DE "Supported Employment" OR DE "Work Adjustment Training" OR DE "Rehabilitation Counseling" OR DE "Rehabilitation Centers" OR DE "Psychosocial Rehabilitation" OR DE "Therapeutic Social Clubs" OR DE "Neuropsychological Rehabilitation" OR DE "Cognitive Rehabilitation" OR DE "Habilitation" OR DE "Self-Care Skills" OR DE "Rehabilitation Centers" OR DE "Sheltered Workshops" OR DE "Rehabilitation" OR DE "Cognitive Rehabilitation" OR DE "Neuropsychological Rehabilitation" OR DE "Neurorehabilitation" OR DE "Occupational Therapy" OR DE "Physical Therapy" OR DE "Psychosocial Rehabilitation" OR DE "Mainstreaming" OR DE "Mainstreaming (Educational)" OR DE "Assistive Technology" OR DE "Self-Help Techniques" OR DE "Self-Management" OR DE "Drug Therapy" OR DE "Socialization" OR DE "Professional Socialization" OR DE "Social Adjustment" OR DE "Social Skills" OR DE "Social Support" OR DE "Behavior Therapy" OR DE "Cognitive Behavior Therapy" OR DE "Acceptance and Commitment Therapy")) (379,141)

S3 S1 OR S2 (140,448)

S2 TI ( ("Intellectual* disab*" OR "intellectual development Disorder*" OR "intellectual and developmental dis*" OR "developmental* disab*" OR "mental* retard*" OR "mental* handicap*" OR "learning disab*" OR Down's OR "Down Syndrome") ) OR AB ( ("Intellectual* disab*" OR "intellectual development Disorder*" OR "intellectual and developmental dis*" OR "developmental* disab*" OR "mental* retard*" OR "mental* handicap*" OR "learning disab*" OR Down's OR "Down Syndrome") )  (115,116)

S1 (DE "Intellectual Development Disorder" OR DE "Down's Syndrome" OR DE "Developmental Disabilities" OR DE "Learning Disabilities" OR DE "Learning Disorders (81,543)

**Cochrane Central Register of Controlled Clinical Trials 7.2.2019**

("Intellectual* disab*" or "intellectual development Disorder*" or "intellectual and developmental dis*" or "developmental* disab*" or "mental* retard*" or "mental* handicap*" or "learning disab*" or Down's or "Down Syndrome") in Record Title AND ("sheltered employment" or "sheltered Work" or employment or employab* or career or workplace or career "Work ability" or "Work capacity" or "ability to Work" or "vocational status" or "vocational adjustment") in Title Abstract Keyword AND (rehabilitat* or treatment* or habilitation or therap* or training or education* or program* or "assistive technolog*" or "assistive devic*" or "self-help devic*" or intervention* or "career counseling" or "social support" or "self management support" or "self care") in Title Abstract Keyword (3)

**Cochrane Database of Systematic Reviews 7.2.2019**

("Intellectual* disab*" or "intellectual development Disorder*" or "intellectual and developmental dis*" or "developmental* disab*" or "mental* retard*" or "mental* handicap*" or "learning disab*" or Down's or "Down Syndrome") in Record Title AND ("sheltered employment" or "sheltered Work" or employment or employab* or "Work ability" or "Work capacity" or "ability to Work" or "vocational status" or "vocational adjustment") in Title Abstract Keyword - with Cochrane Library publication date Between Apr 2016 and Feb 2019, in Cochrane Reviews (Word variations have been searched) (0)

("Intellectual* disab*" or "intellectual development Disorder*" or "intellectual and developmental dis*" or "developmental* disab*" or "mental* retard*" or "mental* handicap*" or "learning disab*" or Down's or "Down Syndrome") in Keyword AND ("sheltered employment" or "sheltered Work" or employment or employab* or "Work ability" or "Work capacity" or "ability to Work" or "vocational status" or "vocational adjustment") in Title Abstract Keyword - with Cochrane Library publication date Between Apr 2016 and Feb 2019, in Cochrane Reviews (Word variations have been searched) (0)

**SocIndex with fulltext 6.2.2019**

S14 S3 AND S6 AND S10 AND S13 (10)
Limiters - Date of Publication: 20160901-20190231

S13 S11 OR S12 (175,544)

S12 TI(CCT OR RCT OR random* OR "clinical trial*" OR "clinical study" OR "controlled clinical" OR "controlled trial*" OR "controlled study" OR "case-control*" OR Cohort OR "prospective study" OR "concurrent study" OR "follow up" OR longitudinal* OR "comparative study" OR "before-after" OR "case stud*" OR "systematic review*" OR meta-analysis OR "qualitative study" OR "qualitative design" OR "qualitative method*" OR "quantitative study" OR "quantitative design*" OR "quantitative method*" OR "validation study" OR "evaluation study" OR "effectiveness study" OR "program evaluation" OR "program* effectiveness" OR "treatment outcome*" OR "outcome* assessment" ) OR AB(CCT OR RCT OR random* OR "clinical trial*" OR "clinical study" OR "controlled clinical" OR "controlled trial*" OR "controlled study" OR "case-control*" OR Cohort OR "prospective study" OR "concurrent study" OR "follow up" OR longitudinal* OR "comparative study" OR "before-after" OR "case stud*" OR "systematic review*" OR meta-analysis OR "qualitative study" OR "qualitative design" OR "qualitative method*" OR "quantitative study" OR "quantitative design*" OR "quantitative method*" OR "validation study" OR "evaluation study" OR "effectiveness study" OR "program evaluation" OR "program* effectiveness" OR "treatment outcome*" OR "outcome* assessment") OR KW(CCT OR RCT OR random* OR "clinical trial*" OR "clinical study" OR "controlled clinical" OR "controlled trial*" OR "controlled study" OR "case-control*" OR Cohort OR "prospective study" OR "concurrent study" OR "follow up" OR longitudinal* OR "comparative study" OR "before-after" OR "case stud*" OR "systematic review*" OR meta-analysis OR "qualitative study" OR "qualitative design" OR "qualitative method*" OR "quantitative study" OR "quantitative design*" OR "quantitative method*" OR "validation study" OR "evaluation study" OR "effectiveness study" OR "program evaluation" OR "program* effectiveness" OR "treatment outcome*" OR "outcome* assessment") (162,614)

S11 DE "CLINICAL trials" OR DE "RANDOMIZED controlled trials" OR DE "QUALITATIVE research" OR DE "QUANTITATIVE research" OR DE "EVALUATION research (Social action programs)" OR DE "FOLLOW-up studies (Medicine)") OR DE "EVENT history analysis" OR DE "COHORT analysis" OR DE "OUTCOME assessment (Social services)" OR DE "SECONDARY analysis" (25,336)

S10 S7 OR S8 OR S9 (124,448)

S9 TI ( ( (("transition to" OR "entry into" OR "entry to" OR entering) N2 (Work OR labo?r OR vocation*)) ) ) OR AB ( ( (("transition to" OR "entry into" OR "entry to" OR entering) N2 (Work OR labo?r OR vocation*)) ) ) OR KW ( ( (("transition to" OR "entry into" OR "entry to" OR entering) N2 (Work OR labo?r OR vocation*)) ) ) (1,384)

S8 TI ( ("sheltered employment" OR "sheltered Work" OR employment OR employab* OR "Work ability" OR "Work capacity" OR "ability to Work" OR "vocational status" OR "vocational adjustment") ) OR AB ( ("sheltered employment" OR "sheltered Work" OR employment OR employab* OR "Work ability" OR "Work capacity" OR "ability to Work" OR "vocational status" OR "vocational adjustment" OR "SCHOOL-to-work transition") OR AB ( ("sheltered employment" OR "sheltered Work" OR employment OR employab* OR "Work ability" OR "Work capacity" OR "ability to Work" OR "vocational status" OR "vocational adjustment") ) OR AB ( ("sheltered employment" OR "sheltered Work" OR employment OR employab* OR "Work ability" OR "Work capacity" OR "ability to Work" OR "vocational status" OR "vocational adjustment" OR "SCHOOL-to-work transition") OR KW ( ("sheltered employment" OR "sheltered Work" OR employment OR employab* OR "Work ability" OR "Work capacity" OR "ability to Work" OR "vocational status" OR "vocational adjustment") ) OR AB ( ("sheltered employment" OR "sheltered Work" OR employment OR employab* OR "Work ability" OR "Work capacity" OR "ability to Work" OR "vocational status" OR "vocational adjustment" OR "SCHOOL-to-work transition" (103,991)

S7 DE "EMPLOYMENT (Economic theory)" OR DE "EMPLOYMENT & education" OR DE "TEMPORARY employment" OR DE "PEOPLE with disabilities -- Employment" OR DE "SELF-employment" OR DE "FULL-time employment" OR DE "SUPPORTED employment" OR DE "EMPLOYABILITY" OR DE "EMPLOYMENT changes" OR DE "SHELTERED workshops" OR DE "LABOR market" OR DE "LABOR supply" OR DE "EMPLOYEE training" OR "CAREER development" (38,029)

S6 S4 OR S5 (648,436)

S5 TI ( (rehabilitat* OR treatment* OR habilitation OR therap* OR training OR education* OR program* OR "assistive technolog*" OR "assistive devic*" OR "self-help devic*" OR intervention* OR "career counseling" OR "social support" OR "self management support" OR "self care" OR "assertiveness training") OR AB ( (rehabilitat* OR treatment* OR habilitation OR therap* OR training OR education* OR program* OR "assistive technolog*" OR "assistive devic*" OR "self-help devic*" OR intervention* OR "career counseling" OR "social support" OR "self management support" OR "self care" or "assertiveness training") OR KW ( (rehabilitat* OR treatment* OR habilitation OR therap* OR training OR education* OR program* OR "assistive technolog*" OR "assistive devic*" OR "self-help devic*" OR intervention* OR "career counseling" OR "social support" OR "self management support" OR "self care" or "assertiveness training") (604,163)

S4 DE "VOCATIONAL rehabilitation" OR "VOCATIONAL guidance" OR DE "VOCATIONAL therapy" OR DE "REHABILITATION" OR DE "TRAINING" OR DE "TREATMENT programs" OR DE "ADJUSTMENT (Psychology)" OR DE "THERAPEUTICS" OR DE "EDUCATIONAL intervention" OR DE "EARLY intervention (Education)" OR DE "INTERVENTION (Social services)" OR DE "HEALTH care intervention (Social services)" OR DE "SOCIAL skills" OR DE "LIFE skills" OR DE "SOCIALIZATION" OR DE "AFFECTIVE education" OR DE "COMMUNITY support" OR DE "SOCIAL support" OR DE "Mentoring" OR DE "SOCIAL Adjustment" OR DE "SOCIALIZATION" OR DE "SUPPORT groups" OR DE "SELF-help devices for people with disabilities" OR DE "AIDS for people with disabilities" OR DE "ASSISTIVE computer technology" OR DE "COMMUNICATION devices for people with disabilities" OR DE "SOCIAL integration" OR DE "SOCIAL Integration Programs" OR DE "MAINSTREAMING in special education" OR DE "SPECIAL education" OR DE "INCLUSIVE education" OR DE "OCCUPATIONAL therapy" OR DE "TREATMENT programs" OR DE "MEDICAL care" OR DE "TRANSITIONAL programs (Education)" OR DE "EMPLOYEE training" OR DE "CAREER education" OR DE "HUMAN services programs" OR DE "COMMUNITY support" (101,773)

S3 S1 OR S2 (28,067)

S2 TI ( ("Intellectual* disab*" OR "intellectual development Disorder*" OR "intellectual and developmental dis*" OR "developmental* disab*" OR "mental* retard*" OR "mental* handicap*" OR "learning disab*" OR Down's OR "Down Syndrome") ) OR AB ( ("Intellectual* disab*" OR "intellectual development Disorder*" OR "intellectual and developmental dis*" OR "developmental* disab*" OR "mental* retard*" OR "mental* handicap*" OR "learning disab*" OR Down's OR "Down Syndrome") ) OR KW ( ("Intellectual* disab*" OR "intellectual development Disorder*" OR "intellectual and developmental dis*" OR "developmental* disab*" OR "mental* retard*" OR "mental* handicap*" OR "learning disab*" OR Down's OR "Down Syndrome") (22,224)

S1 (DE "DEVELOPMENTALLY disabled" OR DE "PEOPLE with disabilities" OR DE "DEVELOPMENTAL disabilities" OR DE "LEARNING disabled persons" OR DE "MENTAL disabilities" OR DE "MENTAL retardation" OR DE "PEOPLE with mental disabilities") (9,589)

**EMBASE 6.2.2019**

1 intellectual impairment/ or "disorders of higher cerebral function"/ or mental deficiency/ or down syndrome/ or learning disorder/ or developmental disorder/ (152458)

2 ("Intellectual* disab*" or "intellectual development Disorder*" or "intellectual and developmental dis*" or "developmental* disab*" or "mental* retard*" or "mental* handicap*" or "learning disab*" or Down's or "Down Syndrome").ti,ab,kw. (103584)

3 1 or 2 (177786)

4 vocational education/ or vocational guidance/ or vocational rehabilitation/ or psychosocial rehabilitation/ or rehabilitation equipment/ or rehabilitation/ or rehabilitation care/ or cognitive rehabilitation/ or rehabilitation medicine/ or rehabilitation center/ or rehabilitation nursing/ or community based rehabilitation/ or socialization/ or early intervention/ or therapy/ or technical aid/ or assistive technology device/ or early childhood intervention/ or intervention study/ or sheltered workshop/ or psychiatric treatment/ or social support/ or self help/ or self care/ or special education/ or education of intellectually disabled/ (1564198)

5 (rehabilitat* or treatment* or habilitation or therap* or training or education* or program* or "assistive technolog*" or "assistive devic*" or "self-help devic*" or intervention* or "career counseling" or "social support" or "self management support" or "self care").ti,ab,kw. (9312295)

6 (rh or th).fs. (1532188)

7 or/4-6 (10502645)

8 exp employment/ or unemployment/ or work capacity/ or workplace/ or job performance/ (140950)

9 ("sheltered employment" or "sheltered Work" or employment or employab* or "Work ability" or "Work capacity" or "ability to Work" or "vocational status" or "vocational adjustment").ti,ab,kw. (72708)

10 (("transition to" or "entry into" or "entry to" or entering) adj3 (Work or labo?r or vocation*)).ti,ab,kw. (1260)

11 or/8-10 (176405)

12 exp clinical trial/ or exp controlled atmosphere/ or controlled clinical trial/ or exp follow up/ or exp cohort analysis/ or exp comparative study/ or exp comparative effectiveness/ or exp longitudinal study/ or exp evaluation study/ or program effectiveness/ or exp outcome assessment/ or exp treatment outcome/ or random sample/ or prospective study/ or exp qualitative analysis/ or exp qualitative research/ or exp quantitative study/ or exp quantitative analysis/ or exp "systematic review"/ (5471625)

13 (CCT or RCT or random* or "clinical trial*" or "clinical study" or "controlled clinical" or "controlled trial*" or "controlled study" or "case-control*" or Cohort or "prospective study" or "concurrent study" or "follow up" or longitudinal* or "comparative study" or "before-after" or "case stud*" or "systematic review*" or meta-analysis or "qualitative study" or "qualitative design" or "qualitative method*" or "quantitative study" or "quantitative design*" or "quantitative method*" or "validation study" or "evaluation study" or "effectiveness study" or "program evaluation" or "program* effectiveness" or "treatment outcome*" or "outcome* assessment").ti,ab,kw. (4304255)

14 or/12-13 (7184119)

15 3 and 7 and 11 and 14 (559)

16 limit 15 to em=201604-201902 (119)

**OTseeker 7.2.2019**

[Diagnosis/Subdiscipline] like 'Intellectual disability' AND [Any Field] like 'employment' AND [Year Published] = '2016' to 2019 (0)

[Diagnosis/Subdiscipline] like 'Intellectual disability' AND [Any Field] like 'employability' AND [Year Published] = '2016' to 2019 (0)

[Diagnosis/Subdiscipline] like 'Intellectual disability' AND [Any Field] like 'labor market' AND [Year Published] = '2016' to 2019 (0)

[Diagnosis/Subdiscipline] like 'Intellectual disability' AND [Any Field] like 'work ability' AND [Year Published] = '2016' to 2019 (0)

[Diagnosis/Subdiscipline] like 'Intellectual disability' AND [Any Field] like 'work capacity' AND [Year Published] = '2016' to 2019 (0)

**PEDRO 6.2.2019**

"intellectual disability" and employ* (1)
down’s and employ* (0)
"down syndrom*" and employ*(0)
"mental retardation" and employ* (0)
"mentally retard*" and employ* (0)
"learning disability*" and employ* (0)
Published since 2016-

**Medic 6.2.2019**

kehitysvamma* "down's" "downin" "kognitiiviset häiriöt" AND työpaik* työllisyys työllist* työelämä* suojatyö* AND kuntou* intervent* ohjelm* terap* 2016 - 2019 (3)

**ERIC (ProQuest) 6.2.2019**

S14 S3 AND S6 AND S10 AND S13 (20)

S13 S10 OR S11 (25,409)

S12 TI((CCT OR RCT OR randomi* OR "clinical trial*" OR "clinical study" OR "controlled clinical" OR "controlled trial*" OR "controlled study" OR "case-control*" OR Cohort OR "prospective study" OR "concurrent study" OR "follow up" OR longitudinal* OR "comparative study" OR "before-after" OR "systematic review*" OR meta-analysis OR "qualitative study" OR "qualitative design" OR "qualitative method*" OR "quantitative study" OR "quantitative design*" OR "quantitative method*" OR "validation study" OR "evaluation study" OR "effectiveness study" OR "program evaluation" OR "program* effectiveness" OR "treatment outcome*" OR "outcome* assessment")) OR AB((CCT OR RCT OR "randomi?ed study" OR "randomi?ed trial" OR "clinical trial*" OR "clinical study" OR "controlled clinical" OR "controlled trial*" OR "controlled study" OR "case-control*" OR Cohort OR "prospective study" OR "concurrent study" OR "follow up" OR longitudinal* OR "comparative study" OR "before-after" OR "systematic review*" OR meta-analysis OR "qualitative study" OR "qualitative design" OR "qualitative method*" OR "quantitative study" OR "quantitative design*" OR "quantitative method*" OR "validation study" OR "evaluation study" OR "effectiveness study" OR "program evaluation" OR "program* effectiveness" OR "treatment outcome*" OR "outcome* assessment")) (8,995)
Applied filters 2016-04-21 - 2019-02-06

S11 SU.EXACT.EXPLODE("Outcomes of Treatment") OR SU.EXACT("Followup Studies") OR SU.EXACT.EXPLODE("Meta Analysis") OR SU.EXACT.EXPLODE("Longitudinal Studies") OR SU.EXACT("Summative Evaluation") OR SU.EXACT("Program Evaluation") OR SU.EXACT("Program Effectiveness") OR SU.EXACT.EXPLODE("Qualitative Research") OR SU.EXACT.EXPLODE("Controlled Environment") OR SU.EXACT.EXPLODE("Comparative Analysis") OR SU.EXACT.EXPLODE("Cohort Analysis") (22,250)
Applied filters 2016-04-21 - 2019-02-06

S10 S7 OR S8 OR S9 (2,393)

S9 TI((("transition to" OR "entry into" OR "entry to" OR entering) NEAR/2 (Work OR labo?r OR vocation*))) OR AB((("transition to" OR "entry into" OR "entry to" OR entering) NEAR/2 (Work OR labo?r OR vocation*))) (38)
Applied filters 2016-04-21 - 2019-02-06

S8 TI(("sheltered employment" OR "sheltered Work" OR employment OR employab* OR "Work ability" OR "Work capacity" OR "vocational status")) OR AB(("sheltered employment" OR "sheltered Work" OR employment OR employab* OR "Work ability" OR "Work capacity" OR "vocational status")) (1,579) Applied filters 2016-04-21 - 2019-02-06

S7 SU.EXACT("Employment Potential") OR SU.EXACT.EXPLODE("Employment") OR SU.EXACT("Employment Experience") OR SU.EXACT("Employment Statistics") OR SU.EXACT("Employment Level") OR SU.EXACT("Part Time Employment") OR SU.EXACT("Employment Qualifications") OR SU.EXACT.EXPLODE("Supported Employment") OR SU.EXACT("Self Employment") OR SU.EXACT("Employment Patterns") OR SU.EXACT("Employment Opportunities") OR SU.EXACT("Labor Market") (1,596)
Applied filters 2016-04-21 - 2019-02-06

S6 S4 OR S5 (56,544)

S5 ti((rehabilitat* OR treatment* OR habilitation OR therap* OR training OR education* OR program* OR "assistive technolog*" OR "assistive devic*" OR "self-help devic*" OR intervention* OR "career counseling" OR "social support" OR "self management support" OR "self care")) OR ab((rehabilitat* OR treatment* OR habilitation OR therap* OR training OR education* OR program* OR "assistive technolog*" OR "assistive devic*" OR "self-help devic*" OR intervention* OR "career counseling" OR "social support" OR "self management support" OR "self care")) (49,464) Applied filters 2016-04-21 - 2019-02-06

S4 SU.EXACT("Assistive Technology") OR SU.EXACT("Vocational Rehabilitation") OR SU.EXACT("Self Management") OR SU.EXACT("Social Support Groups") OR SU.EXACT.EXPLODE("Therapy") OR SU.EXACT("Rehabilitation Counseling") OR SU.EXACT.EXPLODE("Rehabilitation") OR SU.EXACT("Mainstreaming") OR SU.EXACT("Rehabilitation Centers") OR SU.EXACT("Socialization") OR SU.EXACT("Early Intervention") OR SU.EXACT.EXPLODE("Intervention") OR SU.EXACT("Rehabilitation Programs") OR SU.EXACT("Teaching Methods") OR SU.EXACT("Individualized Programs") OR SU.EXACT("Rehabilitation Counseling") OR SU.EXACT("Cooperative Education") OR SU.EXACT("Regular and Special Education Relationship") OR SU.EXACT("Individualized Transition Plans") OR SU.EXACT("Job Training") OR SU.EXACT("Special Education") OR SU.EXACT("Work Experience Programs") OR SU.EXACT("Individualized Education Programs") OR SU.EXACT("Sheltered Workshops") OR SU.EXACT("Individualized Instruction") OR SU.EXACT("Counseling") OR SU.EXACT("Vocational Education") (25,581)
Applied filters 2016-04-21 - 2019-02-06

S3 S1 OR S2 (1,323)

S2 TI(("Intellectual* disab*" OR "intellectual development Disorder*" OR "intellectual and developmental dis*" OR "developmental* disab*" OR "mental* retard*" OR "mental* handicap*" OR "learning disab*" OR Down's OR "Down Syndrome")) OR ab(("Intellectual* disab*" OR "intellectual development Disorder*" OR "intellectual and developmental dis*" OR "developmental* disab*" OR "mental* retard*" OR "mental* handicap*" OR "learning disab*" OR Down's OR "Down Syndrome")) (817)
Applied filters 2016-04-21 - 2019-02-06

S1 SU.EXACT("Mental Retardation") OR SU.EXACT("Mild Mental Retardation") OR SU.EXACT("Moderate Mental Retardation") OR SU.EXACT("Severe Mental Retardation") OR SU.EXACT("Down Syndrome") OR SU.EXACT("Learning Disabilities") (790)
Applied filters 2016-04-21 - 2019-02-06

**PubMed 6.2.2019**

S1 "Developmental Disabilities"[Mesh] OR "Intellectual Disability"[Mesh] OR "Mentally Disabled Persons"[Mesh] Filters activated: Publication date from 2016/04/29 to 2019/02/06 (6276)

S2 (("Intellectual* disab*"[Title/Abstract] OR "intellectual development Disorder*"[Title/Abstract] OR "intellectual[Title/Abstract] AND developmental dis*"[Title/Abstract] OR "developmental* disab*"[Title/Abstract] OR "mental* retard*"[Title/Abstract] OR "mental* handicap*"[Title/Abstract] OR "learning disab*"[Title/Abstract] OR Down's[Title/Abstract] OR "Down Syndrome"[Title/Abstract])) Filters activated: Publication date from 2016/04/29 to 2019/02/06 (2155)

S3 S1 OR S2 (7439)

S4 "Rehabilitation"[Mesh] OR "Early Intervention (Education)"[Mesh] OR "Intervention Studies"[Mesh] OR "Mainstreaming (Education)"[Mesh] OR "Vocational Guidance"[Mesh] OR "Vocational Education"[Mesh] OR "Socialization"[Mesh] OR "Social Support"[Mesh] OR "Sheltered Workshops"[Mesh] OR "Self Care"[Mesh] OR "Self-Help Devices"[Mesh] OR "Remedial Teaching"[Mesh] OR "Rehabilitation, Vocational"[Mesh] OR "Education of Intellectually Disabled"[Mesh] OR "Behavior Therapy"[Mesh] OR "Therapeutics"[Mesh] OR OR "Person-Centred Planning"[Mesh] OR "Education, Special"[Mesh] OR "Rehabilitation Nursing"[Mesh] OR "Rehabilitation of Speech and Language Disorders"[Mesh] OR "Rehabilitation Centers"[Mesh] OR "Physical and Rehabilitation Medicine"[Mesh] OR "Rehabilitation Research"[Mesh] OR "Cognitive Therapy"[Mesh] Filters activated: Publication date from 2016/04/29 to 2019/02/06 (367,203)

S5 (rehabilitat*[Title/Abstract] OR treatment*[Title/Abstract] OR habilitation[Title/Abstract] OR therap*[Title/Abstract] OR training[Title/Abstract] OR education*[Title/Abstract] OR program*[Title/Abstract] OR "assistive technolog*"[Title/Abstract] OR "assistive devic*"[Title/Abstract] OR "self-help devic*"[Title/Abstract] OR intervention*[Title/Abstract] OR "career counseling"[Title/Abstract] OR "social support"[Title/Abstract] OR "self management support"[Title/Abstract] OR "self care"[Title/Abstract]) Filters activated: Publication date from 2016/04/29 to 2019/02/06 (1156756)

S6 S4 OR S5 (1303044)

S7 "Employment"[Mesh] OR "Sheltered Workshops"[Mesh] OR "Work Capacity Evaluation"[Mesh] OR "Employment, Supported"[Mesh] OR "Workplace"[Mesh] Filters activated: Publication date from 2016/04/29 to 2019/02/06 (7633)

S8 (("sheltered employment"[Title/Abstract] OR "sheltered Work"[Title/Abstract] OR employment[Title/Abstract] OR employab*[Title/Abstract] OR "Work ability"[Title/Abstract] OR "Work capacity"[Title/Abstract] OR "ability to Work"[Title/Abstract] OR "vocational status"[Title/Abstract] OR "vocational adjustment"[Title/Abstract] OR "transition into labor"[Title/Abstract] OR "transition into work"[Title/Abstract] OR "transition from school to work"[Title/Abstract])) Filters activated: Publication date from 2016/04/29 to 2019/02/06 (9372)

S9 S7 OR S8 (15064)

S10 "Evaluation Studies" [Publication Type] OR "Comparative Study" [Publication Type] OR "Evaluation Studies as Topic"[Mesh] OR "Cohort Studies"[Mesh] OR "Case-Control Studies"[Mesh] OR "Controlled Before-After Studies"[Mesh] OR "Follow-Up Studies"[Mesh] OR "Clinical Trial" [Publication Type] OR "Validation Studies" [Publication Type] OR "Program Evaluation"[Mesh] OR "Patient Outcome Assessment"[Mesh] OR "Comparative Effectiveness Research"[Mesh] OR "Validation Studies as Topic"[Mesh] OR "Qualitative Research"[Mesh] OR "Treatment Outcome"[Mesh] OR "Prospective Studies"[Mesh] OR "Clinical Trials as Topic"[Mesh] OR "Controlled Clinical Trial" [Publication Type] OR "Pragmatic Clinical Trial" [Publication Type] OR "Meta-Analysis as Topic"[Mesh] OR "Non-Randomized Controlled Trials as Topic"[Mesh] OR "Pragmatic Clinical Trials as Topic"[Mesh] OR "Randomized Controlled Trial" [Publication Type] Filters activated: Publication date from 2016/04/29 to 2019/02/06 (518761)

S11 ((CCT[Title/Abstract] OR RCT[Title/Abstract] OR random*[Title/Abstract] OR "clinical trial*"[Title/Abstract] OR "clinical study"[Title/Abstract] OR "controlled clinical"[Title/Abstract] OR "controlled trial*"[Title/Abstract] OR "controlled study"[Title/Abstract] OR "case-control*"[Title/Abstract] OR Cohort[Title/Abstract] OR "prospective study"[Title/Abstract] OR "concurrent study"[Title/Abstract] OR "follow up"[Title/Abstract] OR longitudinal*[Title/Abstract] OR "comparative study"[Title/Abstract] OR "before-after"[Title/Abstract] OR "case stud*"[Title/Abstract] OR "systematic review*"[Title/Abstract] OR meta-analysis[Title/Abstract] OR "qualitative study"[Title/Abstract] OR "qualitative design"[Title/Abstract] OR "qualitative method*"[Title/Abstract] OR "quantitative study"[Title/Abstract] OR "quantitative design*"[Title/Abstract] OR "quantitative method*"[Title/Abstract] OR "validation study"[Title/Abstract] OR "evaluation study"[Title/Abstract] OR "effectiveness study"[Title/Abstract] OR "program evaluation"[Title/Abstract] OR "program* effectiveness"[Title/Abstract] OR "treatment outcome*"[Title/Abstract] OR "outcome* assessment"[Title/Abstract])) Filters activated: Publication date from 2016/04/29 to 2019/02/06 (573784)

S12 S10 OR S11 (863,842)

S13 S3 AND S6 AND S9 AND S12 (24)

**BASE (Bielefield Academic Search Engine) 7.2.2019**

subj:"intellectual disability" tit:employ* year:[2016 TO 2019] (28)
subj:"intellectual disabilities" tit:employ* year:[2016 TO 2019] (18)

tit:("intellectual disability" "intellectual disabilities" "developmental disability" "developmental disabilities") tit:(employment employability "work capacity" job labor career vocational) tit:(intervention rehabilitation treatment therapy mainstreaming education program technogy assistive device counseling guidance) year:[2016 TO 2019] (20)

tit:("intellectual disability" "intellectual disabilities" "mental handicap" "mentally handicapped" "mental retardation" "mentally retarded" "developmental disability" "developmental disabilities" "downs syndrom*" "mental retardation" "mentally retarded" "intellectual impairment" "intellectually impaired" "learning disability" "learning disabilities") tit:(employment employability "work capacity" job labor career vocational) subj:(intervention rehabilitation treatment therapy mainstreaming education program technology assistive device counseling guidance) year:[2016 TO 2019] (52)

**Google Scholar 7.2.2019**

citations and patents not included in the search

allintitle:"intellectual disability"|"intellectual disabilities"|"intellectually disabled" employment|employability|career|vocational effective|outcomes|interventions|program|rehabilitation (23)

allintitle:"intellectual impairment"|"intellectually impaired"|Down’s employment|employability|career|vocational effective|outcomes|interventions|program|rehabilitation (0)

allintitle:"mental retardation"|"mentally retarded"|"learning disability" employment|employability|career|vocational effective|outcomes|interventions|program|rehabilitation (0)

allintitle:"learning disabilities" employment|employability|career|labor|vocational effective|outcomes|interventions|program|rehabilitation (3)
