# Supplementary material for: The Effectiveness of Rehabilitation Interventions on the Employment and Functioning of People with Intellectual Disabilities: A Systematic Review
Source: J Occup Rehabil. 2019 May 16;29(4):773–802. doi: 10.1007/s10926-019-09837-2 (PMC6838041; doi:10.1007/s10926-019-09837-2)
Supplement: Supplementary file 2 — Supplementary material 2 (DOCX 26 kb) [file 10926_2019_9837_MOESM2_ESM.docx]

Table 6 Quality of included quantitative (RCT, CCT) studies assessed using method of van Tulder [32] with scale: Yes (2), Not known (1), No (0)

| Criteria | Goldberg  et al. [43] | Kilsby and Beyer  [44] |
| --- | --- | --- |
| Was the method of randomization adequate? | 2 | 0 |
| Was the treatment allocation concealed? | 0 | 0 |
| Were the groups similar at baseline in terms of the most important prognostic indicators? | 2 | 2 |
| Was the patient blinded to the intervention? | 0 | 0 |
| Was the care provider blinded to the intervention? | 0 | 0 |
| Was the outcome assessor blinded to the intervention? | 0 | 0 |
| Were co-interventions avoided or similar? | 1 | 1 |
| Was compliance acceptable in all groups? | 2 | 2 |
| Was the dropout rate described and acceptable? | 1 | 2 |
| Was the timing of the outcome assessment similar in all groups? | 2 | 2 |
| Did the analysis include an intention-to-treat analysis? | 2 | 0 |
| Total score (0–22) | 12 | 9 |

Table 7 Quality of included cohort and multimethod studies assessed using method of Wells et al. [36] with scale: 1–3 stars (low quality), 4–6 stars (intermediate quality), and 7–9 stars (high quality).

|  | Selection | Comparability | Outcome/  Exposure | Quality |
| --- | --- | --- | --- | --- |
| Arvidsson et al. [50] | **** | ** | *** | High |
| Beyer and Kaehne [46] | *** | - | * | Intermediate |
| Bouck and Chamberlain [51] | **** | - | * | Intermediate |
| Cimera [47] | **** | ** | *** | High |
| Cimera et al. [49] | **** | ** | *** | High |
| Gray et al. [45] | * | ** | ** | Intermediate |
| Joshi et al. [48] | *** | - | * | Intermediate |
| Sannicandro et al. [52] | **** | ** | *** | High |
| Winsor et al. [59]* | **** | - | ** | Intermediate |

* Multimethod study

**Table 8** Quality of included qualitative studies assessed using CASP method [37] with scale: Yes (2), Partially (1), and No (0).

| Criteria | Alborno and Gaad [56] | Christensen et al. [55] | Devlieger and Trach [57] | Donnelly et al. [58] | Fasching [54] | Hagner and Davies  [53] |
| --- | --- | --- | --- | --- | --- | --- |
| Was there a clear statement of the aims of the research? | 2 | 1 | 1 | 1 | 2 | 2 |
| Was a qualitative methodology appropriate? | 2 | 2 | 2 | 2 | 2 | 2 |
| Was the research design appropriate to address the aims of the research? | 2 | 2 | 2 | 2 | 2 | 1 |
| Was the recruitment strategy appropriate to the aims of the research? | 2 | 2 | 2 | 2 | 1 | 1 |
| Were the data collected in a way that addressed the research issue? | 2 | 2 | 2 | 2 | 2 | 2 |
| Was the possible researcher effect on the results adequately considered? | 2 | 1 | 2 | 2 | 2 | 0 |
| Were ethical issues taken into consideration? | 2 | 2 | 2 | 2 | 1 | 0 |
| Was the data analysis sufficiently rigorous? | 2 | 2 | 2 | 2 | 2 | 1 |
| Was there a clear statement of findings? | 2 | 2 | 2 | 1 | 2 | 1 |
| How valuable is the research? | 2 | 2 | 2 | 1 | 2 | 2 |
| Total score (out of 20) | 20 | 18 | 19 | 17 | 18 | 12 |

**Table 9** Quality assessment of case studies using JBI (Joanna Briggs Institute) method [39] with scale Yes (1), No (2), Unclear (3), Not applicable (4).

| Criteria | Allen et al. [76] | Aspinall [62] | Bennet et al. [72] | Carson et al. [71] | Chang et al. [74] | Devlin [75] | Dotson et al. [77] | Furniss et al. [69] | Gilson and Carter [79] | Grossi et al. [60] | Ham et al. [64] | Jarhag et al. [63] | Kemp and Carr [67] | McGlashing-John-son et al. [70] | McMahon et al. [78] | Renzaglia et al. [65] | Simmons and Flexer [66] | Taber et al. [68] | Van Laarhoven et al. [80] | Wehman et al. [61] | West and Patton [73] |
| --- | --- | --- | --- | --- | --- | --- | --- | --- | --- | --- | --- | --- | --- | --- | --- | --- | --- | --- | --- | --- | --- |
| Were the demographic characteristic of a person with ID clearly described? | 1 | 1 | 1 | 1 | 1 | 1 | 1 | 1 | 1 | 1 | 3 | 1 | 1 | 1 | 1 | 1 | 1 | 1 | 1 | 1 | 1 |
| Was the history of the person with ID clearly described and presented in the timeline? | 1 | 2 | 1 | 1 | 2 | 1 | 1 | 1 | 1 | 1 | 1 | 1 | 1 | 1 | 1 | 1 | 1 | 1 | 1 | 1 | 1 |
| Was the current clinical condition of the person with ID clearly described? | 1 | 1 | 1 | 1 | 1 | 1 | 1 | 2 | 1 | 1 | 1 | 1 | 1 | 1 | 1 | 1 | 1 | 1 | 1 | 1 | 1 |
| Were diagnostic tests or assessment methods and the 1results clearly described? | 1 | 2 | 1 | 1 | 2 | 1 | 1 | 2 | 1 | 1 | 1 | 2 | 1 | 1 | 1 | 1 | 1 | 2 | 1 | 1 | 1 |
| Was the intervention(s) or treatment procedure(s) clearly presented? | 1 | 2 | 1 | 1 | 1 | 1 | 1 | 1 | 1 | 1 | 1 | 2 | 1 | 1 | 1 | 1 | 1 | 1 | 1 | 1 | 1 |
| Was the post-intervention clinical condition clearly presented? | 1 | 1 | 1 | 1 | 1 | 1 | 1 | 1 | 1 | 1 | 1 | 1 | 1 | 1 | 1 | 1 | 1 | 1 | 1 | 1 | 1 |
| Were adverse events (harms) or unanticipated events described? | 2 | 3 | 2 | 2 | 2 | 2 | 2 | 2 | 2 | 1 | 1 | 1 | 2 | 2 | 1 | 2 | 2 | 2 | 2 | 1 | 2 |
| Does the case report provide takeaway lessons? | 1 | 1 | 1 | 1 | 1 | 1 | 1 | 1 | 1 | 1 | 1 | 1 | 1 | 1 | 1 | 1 | 1 | 1 | 1 | 1 | 1 |
